# BEGREM: A Mechanistic Bioelectrochemical Model Coupling Gut Microbial Metabolism

**DOI:** 10.3390/microorganisms14102187

**Published:** 2026-09-30

**Authors:** James Torres-Peralta, Josue Duarte-Cuesta, Ronny Amaguay-Gomez, María Román-Manzano, Samuel Valle-Asan

**Affiliations:** 1 Faculty of Health Sciences and Social Services, Universidad Estatal de Milagro, Milagro 091706, Ecuador; 2 Faculty of Science and Engineering, Universidad Estatal de Milagro, Milagro 091706, Ecuador; jduartec3@unemi.edu.ec (J.D.-C.); ramaguayg@unemi.edu.ec (R.A.-G.); mromanm2@unemi.edu.ec (M.R.-M.)

**Keywords:** colonic microbiota, microbial metabolism, extracellular electron transfer, redox potential, bioelectrochemistry, reducing equivalents, mechanistic modeling, BEGREM

## Abstract

Colonic microbial metabolism emerges from coupled substrate utilization, cross-feeding, redox regulation, extracellular electron transfer (EET), transient reducing-equivalent storage, and spatial transport. We developed BEGREM, a data-constrained mechanistic bioelectrochemical framework that integrates these processes while treating current as a composite observable rather than a direct surrogate of total metabolism. Public data from 22 studies comprised 103,320 current-voltage-power points, 3996 cyclic-voltammetry points, 213 impedance points, and 99 quality-controlled polarization curves. The archived full-BEGREM analysis yielded a median held-out RMSE of 12.4 mV. Independent reconstruction reproduced this scale at 12.147 mV (95% bootstrap: 10.406–14.736 mV) and improved 81/99 curves versus the recoverable Ohmic/Quadratic comparator set, with median ΔRMSE=7.700 mV (95% interval: 4.695–18.175; $p=7.20\times 10^{-11}$). Full BEGREM also showed lower AICc in 95/99 curves. OCP-repolarization identified $Q_{max}=3.575$ mC and $k_{ch}=0.02063s^{-1}$ with profile-likelihood 95% intervals of 2.302–5.023 mC and 0.00634–0.10633 s^−1^. Study-held-out transfer produced RMSEs of 75.94 and 104.15 mV, indicating substantial cross-architecture penalties. BEGREM therefore provides a reproducible mechanistic observation framework with strong within-curve and module-level support, while colon-specific calibration remains necessary.

## 1. Introduction

The human colon is a densely populated anaerobic ecosystem in which dietary and host-derived substrates are transformed through networks of microbial fermentation and cross-feeding. The resident microbiota contributes materially to nutrient recovery and host physiology [1], while acetate, propionate, butyrate, lactate, succinate, and related fermentation intermediates form an interconnected metabolic network rather than a collection of independent end products [2,3]. Dietary fiber is a principal precursor of short-chain fatty acids (SCFAs) [4], long-term dietary composition reshapes microbial community function [5], and host-derived mucins provide an additional carbon and nitrogen reservoir for specialized degraders [6]. This metabolic activity occurs across the structured colonic mucus barrier, which spatially separates dense luminal microbial populations from the epithelium and thereby establishes distinct reaction zones [7].

A second organizing dimension is redox state. Oxygen availability falls sharply toward the anaerobic lumen, while pH, hydrogen partial pressure, and oxidation-reduction potential (ORP) vary with microbial consumption, fermentation stoichiometry, diffusion, and host transport [8]. Fecal redox and pH have consequently been proposed as functional descriptors of microbiome state because they covary with metabolite profiles and community composition [9]. These observations imply that a model restricted to substrate concentration and biomass cannot fully encode the physicochemical boundary conditions under which colonic metabolism operates.

Extracellular electron transfer provides a complementary window on reducing-equivalent disposal. The abundant butyrate-producing commensal *Faecalibacterium prausnitzii* can exploit extracellular flavin/thiol shuttling at oxic-anoxic interfaces [10], and mechanistic analysis has specifically implicated riboflavin in this electron-transfer phenotype [11]. Hydrodynamic chronoamperometry further demonstrated saturable dependence of *F. prausnitzii* electroactivity on both substrate and riboflavin availability [12]. Flavin-based EET is not restricted to this organism: analogous mechanisms occur across phylogenetically diverse gram-positive bacteria [13], EET has been detected directly in mammalian-gut settings [14], and current reviews now recognize extracellular electron exchange as a potentially relevant ecological trait in plant and animal microbiomes [15]. Together, these studies justify evaluating EET as a measurable functional process without assuming that all colonic microorganisms are constitutive electrode-respiring exoelectrogens.

Existing experimental and computational platforms capture important components of this biology but remain fragmented. Anaerobic intestine-on-chip systems can sustain complex human gut communities adjacent to oxygenated epithelium [16], and anoxic-oxic interface chips have demonstrated stable long-term co-culture of obligate anaerobes with intestinal cells [17]. Recent intestinal–organ–chip frameworks further reproduce flow, tissue interfaces, and oxygen gradients relevant to host–microbiome interactions [18]. In parallel, metabolic models of the gut microbiota increasingly resolve species-specific metabolic exchange and cross-feeding, although redox observability and transient electron storage remain incompletely represented [19]. Spatially explicit computational environments such as Virtual Colon extend this direction by representing lumen, mucus, epithelium, transport, and metabolic interactions in a common domain [20]. The unresolved opportunity is therefore not to recast the colon as a microbial fuel cell, but to use bioelectrochemical observables as additional constraints on a mechanistic model of colonic microbial metabolism.

BEGREM was formulated to address this gap by mapping the native colonic cascade onto a minimal set of measurable and latent state variables. Figure 1 summarizes the conceptual correspondence: the left half of Figure 1 depicts the native sequence from fermentable substrates through microbial functional guilds and metabolite production under local redox gradients, whereas the right half depicts the BEGREM representation in which reducing-equivalent generation feeds the dynamic state, direct EET (DET), mediated EET (MET), redox feedback, and transport. The sensing electrode is therefore an observation interface for microbial redox behavior, not the physiological endpoint of the model.

On this basis, the study addressed four mechanistic hypotheses and one integrative prediction task. First, distinct EET routes should produce resolvable electrochemical signatures that justify separate DET and MET terms. Second, ORP should constrain microbial metabolic behavior beyond a purely constant response, although its independent contribution must be tested against substrate covariation. Third, history-dependent charge accumulation and release should require an effective reducing-equivalent state, $Q_{m}$, rather than a memoryless current-voltage relationship. Fourth, transport-generated spatial gradients should require a spatial extension whenever local measurements are available. Finally, the integrated architecture should improve held-out prediction relative to progressively simpler electrical and polarization models without relying solely on additional degrees of freedom.

The contribution tested here is therefore not the proposition that the colon behaves as a microbial fuel cell, nor that electrical current uniquely measures metabolism. Rather, BEGREM contributes a single mechanistic architecture that couples substrate conversion, redox-dependent metabolic allocation, history-dependent reducing-equivalent storage, DET/MET flux classes, and spatial transport to experimentally accessible observables. This architecture generates falsifiable predictions at electrical, redox, transient, and spatial levels and provides a defined route for future colon-specific calibration instead of assuming physiological equivalence a priori.

## 2. Materials and Methods

### 2.1. Study Design and Public Data Sources

This work was designed as a secondary data mechanistic modeling study. No new human or animal samples were collected. Studies were eligible when they (i) reported peer-reviewed experimental bioelectrochemical, microbial-metabolic, redox, transient-charge, transport, spatial-profile, or microbiome measurements; (ii) provided numerical data in the publication, Supplementary Files, or a persistent public repository; and (iii) contributed a measurable constraint to at least one BEGREM state, flux, observation, boundary condition, or evidence layer. Studies used only to establish canonical equations or statistical procedures were separated from the 22-study experimental evidence base. The selection strategy was intentionally mechanistic rather than meta-analytic: heterogeneous datasets were retained because they interrogated different terms of the proposed state-space model.

#### Evidence Selection, Provenance, and Scope

This study is a mechanistic secondary data integration rather than a systematic review or meta-analysis. Candidate sources were retained only when the numerical evidence was traceable to the publication, Supplementary Files, or a persistent repository and when the source constrained a predefined BEGREM component. Sources lacking recoverable quantitative information, duplicate datasets, purely narrative reports, and studies that did not map to a state, flux, observation, or boundary condition were not used as experimental evidence. The 22-study set, therefore, represents a mechanism-oriented evidence architecture, not an estimate of a pooled biological effect. Each source is interpreted according to its evidentiary role (calibration, comparative reference, mechanistic support, or physiological anchoring), and non-colonic systems are not treated as direct surrogates of the human colon.

The final evidence base comprised 22 public experimental sources spanning metabolic response, controlled redox, EET/electrochemistry, intermittent charge storage, forced flow, spatial profiling, and limited taxonomic identification. To avoid presenting repository addresses as if they were bibliographic citations, Table 1 reports each source using the MDPI numerical reference assigned to the corresponding article or dataset. The persistent repository DOI or accession used for reproducibility is compiled in Supplementary Table S1 (source-data manifest), while the curated study-level metadata used to map each source to BEGREM modules are provided in Supplementary Table S2. The Reference column links each evidence source to its bibliographic record, while persistent repository identifiers remain in Supplementary Table S1 for auditability.

### 2.2. Data Harmonization, Experimental Units, and Quality Control

Raw measurements were retained in source units for traceability and converted to SI-compatible units only when required by a fitted equation. The experimental hierarchy was preserved explicitly as study → reactor → condition → curve → point; consequently, dense time series and polarization points were never treated as independent biological replicates. This rule was particularly important for the rainwater electrochemical dataset [22], whose original Origin project files were parsed at curve level. Calibration datasets were selected because they directly constrained mediator-dependent transfer [21], controlled-redox metabolism [26], forced-flow behavior [33], transient charge storage [36], or spatial microsensor gradients [37], while the remaining sources provided within-dataset predictive evaluation, comparative evidence, mechanistic support, conversion/transport evidence, or abiotic controls [23,24,25,27,28,29,30,31,32,33,34,35,38,39,40,41,42]. The Supplementary Material archive provides the original-value workbook and the exact point-level I-V-P, CV, EIS, and microbial-identification files available in the working dataset; variable names, symbols, source units, SI harmonization, and model mapping are documented in Supplementary Table S3.

The 14 microbial records retained from the rainwater dataset are treated only as taxonomic identifications. They do not constitute a complete abundance matrix and are not used to infer alpha diversity, beta diversity, community succession, or species-resolved contributions to BEGREM current. Community-level validation will require independent abundance-resolved 16S rRNA gene, metagenomic, or absolute-quantification data.

Figure 2 summarizes the evidence architecture and quality-control logic. Figure 2A maps complete, partial, and unavailable information across mechanistic domains; Figure 2B summarizes non-exclusive evidence-category assignments across the 22 public sources (a study may contribute to more than one mechanistic category); Figure 2C preserves the nested experimental hierarchy and identifies the 99 usable electrochemical curves; Figure 2D reports the quantitative richness of the integrated electrochemical data. Specifically, 103,320 current-voltage-power points, 3996 cyclic-voltammetry (CV) points, 213 electrochemical-impedance-spectroscopy (EIS) points, and 14 microbial identifications were retained. Of 110 extracted polarization segments, 100 were unique by curve hash, and 99 were model-eligible after exclusion of one scale inconsistency. These counts document data sufficiency but are not interpreted as independent replicate numbers. The curve-level hashes, duplicate groups, and scale-quality flag underlying this filtering are reported in Supplementary Table S5.

### 2.3. BEGREM State Variables and Mechanistic Formulation

BEGREM was implemented as a coupled state-space framework in which microbial metabolism generates reducing equivalents that can be allocated to biomass and metabolic products, retained transiently in an effective reservoir, or released through extracellular electron-transfer pathways. The formulation builds sequentially on canonical substrate-limited microbial kinetics [43], conduction-based biofilm-anode descriptions [44], reaction-transport biofilm modeling [45], dynamic population balances in continuous bioelectrochemical reactors [46], Butler–Volmer–Monod coupling [47], standardized MFC measurement conventions [48], and microbial-electrosynthesis concepts in which electron flow directly constrains microbial conversion [49]. Electrochemical potentials and mixed redox states follow conventional electrochemical thermodynamics [50], while the biological interpretation of extracellular electron exchange is informed by contemporary EET mechanisms [51] and EIS analysis of bioelectrochemical interfaces [52]. Against this established basis, the BEGREM-specific contribution is the coupling of these processes to colonic metabolic observables and to the effective history-dependent state. Table 2 summarizes the principal states and observables. Parameter admissibility bounds and the seed values used to initialize numerical estimation are reported in Supplementary Tables S4 and S4b, respectively; the executable implementation of Equations (1)–(14) is provided in BEGREM_model_core.py.

#### 2.3.1. Microbial Metabolism and Mass Balances

The microbial conversion rate was represented as a saturable substrate term multiplied by a saturable oxidized-mediator term and bounded environmental response functions. The first saturation is rooted in Monod kinetics [43], whereas mediator dependence is supported by the experimentally observed substrate- and riboflavin-dependent electroactivity of *F. prausnitzii* [12]. Their multiplicative coupling is a BEGREM-specific constitutive hypothesis rather than a universal published rate law. The rate $r_{M}$ is defined in Equation (1).

(1) $$r_{M}=k_{cat}X\;\frac{S}{K_{S}+S}\;\frac{M_{ox}}{K_{M}+M_{ox}}\;f_{pH}f_{ORP}f_{O_{2}}$$

In Equation (1), $k_{cat}$ is the apparent catalytic rate constant, $K_{S}$ and $K_{M}$ are half-saturation constants, and $f_{pH}$, $f_{ORP}$, and $f_{O_{2}}$ are bounded dimensionless environmental modifiers. Substrate conservation and active-biomass dynamics are then represented by Equations (2) and (3), respectively.

The core implementation does not impose a universal logistic, Hill, or polynomial response law for $f_{pH}$, $f_{ORP}$, or $f_{O_{2}}$. Each modifier is constrained to the interval [0,1] and is supplied from source-specific calibration or experimental conditions; when no independent environmental modifier is available, the corresponding factor is set to 1.0. Thus, Equation (1) is evaluable for a specified dataset but does not claim to predict pH-, ORP-, or $O_{2}$-response functions de novo without calibration. The controlled-ORP analysis in Section 2.4 and Section 3.2 is therefore an independent diagnostic of redox relevance and identifiability rather than a universal definition of $f_{ORP}$ (Supplementary Table S13).

(2) $$\frac{dS}{dt}=D\left(S_{in}-S\right)-\frac{r_{M}}{Y_{M/S}}$$

(3) $$\frac{dX}{dt}=\left(\mu -k_{d}-D\right)X\;\;$$

Equations (2) and (3) reduce to batch balances when D=0 and retain dilution when D>0. This structure is consistent with established continuous bioelectrochemical population models [46], but BEGREM treats the resulting microbial state as an upstream determinant of redox observables rather than as a reactor-performance endpoint.

#### 2.3.2. Generation, Storage, and Partitioning of Reducing Equivalents

To conserve electron equivalents across the metabolic and electrochemical layers, the molar rate $r_{M}$ was converted to an electrical generation rate using Faraday conservation, as expressed in Equation (4).

(4) $$I_{gen}=n_{e}FV_{R}r_{M}$$

In Equation (4), ne is the electron stoichiometry, F is Faraday’s constant, and VR is the reactive volume. BEGREM then introduces $Q_{m}$ as an effective state that accumulates when reducing-equivalent generation exceeds measured EET and drains through direct transfer, mediator reduction, and other sinks. This balance is given by Equation (5).

(5) $$\frac{dQ_{m}}{dt}=I_{gen}-I_{DET}-I_{Mred}-k_{L}Q_{m}$$

Equation (5) is the central dynamic extension of BEGREM. Its purpose is not to assign all stored charge to one molecular carrier, but to encode experimentally observable history dependence. Intermittent-potential bioanode experiments showed recoverable charge accumulation during open-circuit intervals and transient release after repolarization [36]; $Q_{m}$ provides the minimal latent state required to represent that memory while retaining dimensional conservation in coulombs.

$Q_{m}$ is defined as an effective inventory of kinetically accessible microbial-reducing equivalents expressed as charge equivalents (C). It is not assigned to a unique molecule or intracellular compartment. The state may aggregate contributions from reduced intracellular cofactors, membrane redox components, redox-active extracellular matrix components, and soluble mediators only to the extent that these pools remain kinetically available for subsequent electron transfer. Consequently, evidence for the necessity of a history-dependent state is evaluated separately from molecular identification and from practical identifiability of the individual recharge and discharge parameters.

#### 2.3.3. Direct and Mediated Extracellular Electron Transfer

Direct extracellular electron transfer was represented by a Butler–Volmer-type kernel modulated by the instantaneous availability of $Q_{m}$. The electrode-transfer component follows the mechanistic logic of Butler–Volmer–Monod bioanode models [47], while the $Q_{m}$ saturation term is specific to BEGREM. The resulting DET current is defined in Equation (6).

(6) $$I_{DET}=A_{A}j_{0,D}\frac{Q_{m}}{K_{Q}+Q_{m}}\left[exp\left(\frac{\alpha_{D}nF\eta_{D}}{RT}\right)-exp\left(-\frac{\left(1-\alpha_{D}\right)nF\eta_{D}}{RT}\right)\right]$$

For the DET kernel, $\eta_{D}$ is the electrode overpotential relative to the effective equilibrium potential of the direct-transfer pathway, $\eta_{D}=E_{A}-E_{eq,D}$, using a common reference electrode scale and the sign convention of Equation (6). The electron number n and transfer coefficient $\alpha_{D}$ are dimensionless electrochemical parameters; all potential terms entering the exponential kernel must use the same reference frame.

Transfer from the $Q_{m}$ reservoir to an oxidized soluble mediator is represented by the saturating allocation law in Equation (7).

(7) $$I_{Mred}=k_{M}Q_{m}\frac{M_{ox}}{K_{M,Q}+M_{ox}}$$

The reduced mediator pool is subsequently constrained by conservation between microbial reduction, electrode oxidation, and hydraulic exchange, giving Equation (8).

(8) $$\frac{dM_{red}}{dt}=\frac{I_{Mred}-I_{MET}}{n_{M}FV_{R}}+D(M_{feed}-M_{red})$$

For readability in Equation (8), $M_{feed}$ denotes the influent reduced-mediator concentration and is identical to the source-specific $M_{red,in}$ quantity used in the implementation. In Equations (1) and (7), $M_{ox}$ is an explicit oxidized-mediator input rather than an unreported fitted state. When a finite total mediator inventory $M_{T}$ is independently known, mediator closure can be imposed algebraically as $M_{ox}=M_{T}-M_{red}$; otherwise, $M_{ox}$ is fixed to the experimentally imposed oxidized-mediator level for the corresponding source condition. The current core implementation, therefore, requires $M_{ox}$ (or $M_{T}$ together with $M_{red}$) to be supplied explicitly and does not infer an unobserved total mediator pool (Supplementary Table S13).

The mediator-to-electrode current is represented by Equation (9), which retains Butler–Volmer dependence while limiting flux by the available reduced-mediator pool.

For MET, $\eta_{M}=E_{A}-E_{eq,M}$ is defined analogously using the effective equilibrium potential of the mediator-electrode couple. $E_{A}$ and $E_{eq,M}$ must be expressed in the same reference-electrode frame; $\alpha_{M}$ and n retain their conventional charge-transfer meanings.

(9) $$I_{MET}=A_{A}j_{0,M}\frac{M_{red}}{K_{M,E}+M_{red}}\left[exp\left(\frac{\alpha_{M}nF\eta_{M}}{RT}\right)-exp\left(-\frac{\left(1-\alpha_{M}\right)nF\eta_{M}}{RT}\right)\right]$$

Retaining DET and MET as distinct terms is biologically and experimentally motivated. Gut-associated flavin shuttling and gram-positive EET support mediated pathways [10,11,12,13,14,15], while redox-mediated flow experiments exhibit large pathway-dependent changes in charge-transfer resistance and electrochemical response [21]. Equations (6)–(9) therefore partition electron disposal mechanistically rather than collapsing all current into a single empirical coefficient.

Experimentally, DET- and MET-dominant behavior is interpreted operationally rather than as a uniquely molecular decomposition of total current. Evidence for mediated transfer includes redox-active soluble species, mediator-addition or removal responses, characteristic voltammetric features, and mediator-dependent changes in charge-transfer resistance; DET support requires electrode-associated transfer that persists without an added soluble shuttle. These criteria support functional discrimination of electron-transfer routes, but BEGREM does not claim that Equations (6)–(9) uniquely resolve the molecular fraction of DET and MET in an unperturbed human colonic community.

#### 2.3.4. Bioelectrochemical Observation and Redox Closure

Measured current was not equated directly with microbial metabolism because the observable can include abiotic electrocatalysis, electrode background, and measurement error. The observation layer, therefore, combines DET, MET, and an explicit abiotic contribution, as formalized in Equation (10).

Accordingly, electrical current is treated as a composite bioelectrochemical observable conditional on the microbial redox state, electrode interface, mediator availability, abiotic catalysis, and measurement conditions. It is not used as a universal or one-to-one surrogate for total microbial metabolic rate, SCFA production, community abundance, or host physiology.

(10) $$I_{obs}=I_{DET}+I_{MET}+I_{abiotic}+\varepsilon_{I}$$

The $I_{abiotic}$ term in Equation (10) is necessary because trace metals and medium components can form in situ electrocatalysts and substantially alter measured current independently of microbial activity [34]. Redox couples were related to formal potential using the Nernst relation in Equation (11) [50].

(11) $$E_{i}={E_{i}^{0}}^{\prime }+\frac{RT}{n_{i}F}ln\left(\frac{a_{Ox,i}}{a_{Red,i}}\right)$$

Because ORP in a chemically complex microbial system reflects multiple simultaneous redox couples, the measured mixed potential was closed by the zero-net-current condition in Equation (12).

(12) $$\sum_{i=1}^{N}j_{i}\left(E_{ORP}\right)=0\;\;$$

Equation (12) prevents ORP from being treated as a simple linear surrogate for current. Its use is consistent with spatial microsensor evidence showing simultaneous variation in ORP, pH, $H_{2}$, and electric potential across porous bioelectrochemical microenvironments [37].

#### 2.3.5. Spatial Transport Extension

Initial model fitting used a zero-dimensional representation because most curated datasets reported bulk concentrations or whole-cell electrochemical responses. When positional measurements were available, BEGREM was extended spatially using the standard advection–diffusion–reaction balance in Equation (13).

(13) $$\frac{\partial C_{i}}{\partial t}+u\frac{\partial C_{i}}{\partial x}=D_{i}\frac{\partial^{2}C_{i}}{\partial x^{2}}+R_{i}$$

In Equation (13), *u* is the local advective velocity, $D_{i}$ is the effective diffusion coefficient, and $R_{i}$ is the net production-consumption term for species *i*. The extension is constrained by forced-flow experiments [33] and microsensor profiling [37], which demonstrate that local $H_{2}$, pH, ORP, and electric potential can diverge from bulk measurements.

The transport coefficients used in this formulation should therefore be interpreted as BES-derived or generic effective parameters unless a colon-specific value is independently available. Equation (13) defines the transport-reaction architecture; it does not establish colon-specific calibration of u or $D_{i}$.

### 2.4. Model Hierarchy, Fitting, and Evidence Evaluation

Model comparison was designed to test mechanistic necessity rather than in-sample flexibility. For polarization model comparison, BEGREM was compared with four progressively richer conventional alternatives: an Ohmic model, a quadratic empirical model, a Tafel–Ohmic model, and an activation–Ohmic–concentration (AOC) model. Two BEGREM observation variants were evaluated: the full latent-state formulation and a parsimonious $Q_{m}$-depletion form used when recharge-related parameters were not identifiable from polarization data alone. For each of 99 eligible curves, the first 70% of the current trajectory was used for parameter estimation, and the final 30% high-current region was reserved for forward prediction.

The 70/30 procedure is interpreted strictly as within-curve forward extrapolation rather than study-level external validation. Cross-study transfer was evaluated with two complementary two-fold S02 ↔ S03 analyses using the independent point-level polarization datasets of Amen et al. [22] and Serra [23]. First, an architecture-normalized common-shape LOSO analysis expressed current as $I/I_{max}$ and voltage as $V/V_{OCV}$, with complete curves from one study used for shape estimation, and the other study held out in its entirety (Supplementary Table S16). Second, the reconstructed five-parameter full polarization layer described below was fitted on one study and transferred to the other on a fixed 101-point sweep-fraction grid, preserving complete curves as the uncertainty unit (Supplementary Table S19). These analyses quantify architecture transfer and are reported separately from the archived within-curve comparison.

The curve-level full-BEGREM objects required for reproducible analysis were regenerated through a frozen de novo reconstruction designated BEGREM MODEL complete. The reconstruction used the original S02 point-level polarization curves and the Table S5 quality-control hashes without back-calculation from archived aggregate metrics. The historical split design was preserved: the first ⌊0.70N⌋ points of each eligible trajectory were used for estimation, and the final 30% were held out. The recovered reduced full parameterization contains five parameters ($Q_{m,0}$, $k_{d}$, $k_{r}$, a, b). Current was scaled within the curve as $x_{t}=\frac{max\left(I_{t},0\right)}{{max}_{t}\left[max\left(I_{t},0\right)\right]}$, the latent state was updated as $q_{t+1}=\left(1-k_{d}x_{t}-k_{r}\right)q_{t}+k_{r}$, and voltage was observed as ${\hat{V}}_{t}=aq_{t}-bx_{t}$. The parsimonious reconstruction fixes $q_{0}=1$ and $k_{r}=0$. This frozen closure reconstructs the reduced polarization layer from preserved raw data but is not claimed to reproduce the unavailable legacy optimizer state byte-for-byte. The archived 12.4 mV, 82/99, paired $p=6.27\times 10^{-11}$, and median ΔAICc = 2777 were never used as fitting targets (Supplementary Tables S17 and S18).

For the redox module, constant-response, substrate-only, and ORP-only models were retained from the archived leave-one-condition-out analysis. A separate source-level reanalysis tested whether ORP and substrate effects could be identified independently using the four controlled steady states reported by Arbter et al. [26]. The reconstructed design comprised ORP values of −462, −416, −337, and −250 mV and corresponding residual-glycerol concentrations of 7.65, 13.73, 22.75, and 31.12 g L^−1^. Constant, ORP-only, substrate-only, and joint ORP-plus-substrate linear models of 1,3-propanediol yield were evaluated by leave-one-condition-out prediction. Predictor dependence was quantified using Pearson correlation, the variance inflation factor $VIF=\frac{1}{1-r^{2}}$, and the condition number of the standardized predictor matrix. This source-level diagnostic is reported separately from the archived normalized redox outputs in Supplementary Table S6 because the original redox fit objects were not retained; the additional diagnostics are provided in Supplementary Table S10.

Evidence is separated into five categories: (i) parameter or boundary-condition calibration, (ii) mechanistic support, (iii) within-dataset predictive evaluation, (iv) physiological anchoring against gut-relevant measurements, and (v) colon-specific physiological validation. Only the first four categories are addressed by the present evidence base; category (v) requires an independent gut-mimetic or in-human experimental platform.

The core implementation supports a scaled weighted-residual objective, defined in Equation (14), for heterogeneous endpoints. The objective family follows the form implemented in BEGREM_model_core.py and is distinct from the archived aggregate model-comparison outputs.

(14) $$L\left(\theta \right)=\sum_{k}\sum_{t}w_{k}{\left[\frac{y_{k}\left(t\right)-{\hat{y}}_{k}\left(t|\theta \right)}{\sigma_{k}}\right]}^{2}$$

In Equation (14), θ denotes the fitted parameter vector, $w_{j}$ is an optional non-negative endpoint weight, $\sigma_{j}$ is a positive endpoint-specific residual scale, $y_{j}$ is the observation, and ${\hat{y}}_{j}$ is the model prediction. The executable weighted_loss routine uses $w_{j}=1$ when weights are not supplied. The historical source-specific $w_{j}$ and $\sigma_{j}$ settings used in the archived integrated fitting objects were not retained, so the historical likelihood trajectory remains provenance rather than a fully recoverable object. AICc follows Hurvich and Tsai [53]. For the de novo 99-curve reconstruction, training-region AICc was computed directly from the retained SSE as $nln\left(SSE/n\right)+2k+\frac{2k\left(k+1\right)}{n-k-1}$. The primary AICc audit restricts the curve-specific classical comparator to the unambiguously recoverable Ohmic and quadratic forms; a secondary sensitivity additionally includes de novo Tafel-like and canonical AOC reconstructions. This separation prevents uncertain legacy comparator parameterizations from being presented as exact historical objects (Supplementary Tables S8, S9 and S18–S22).

For the reconstructed full and parsimonious models, the complete 99-curve held-out RMSE distribution, point predictions, fitted parameters, training SSE, residual diagnostics, and curve-wise AICc were retained. Descriptive uncertainty for the median full-BEGREM RMSE and paired ΔRMSE was estimated with 20,000 nonparametric resamples of complete curves using fixed seed 20260922. Dependence sensitivity was assessed by resampling recoverable Book_ID clusters as intact units. The primary paired predictive audit defines the comparator on each curve as $min\left({RMSE}_{Ohmic},{RMSE}_{Quadratic}\right)$; the secondary four-model sensitivity additionally includes reconstructed Tafel-like and AOC alternatives. Residual bias, MAE, lag-1 residual correlation, leave-one-curve influence, and leave-one-Book_ID influence were computed to characterize stability and the dependence structure underlying point-wise likelihood calculations. The reconstructed S02 ↔ S03 full-layer LOSO used $x=I/I_{max}$ and $y=V/V_{OCV}$ on the fixed 101-point grid so that the discrete latent-state update was not confounded by acquisition-point density (Supplementary Tables S18–S22).

Practical $Q_{m}$ identification was performed from the authoritative Pereira et al. [36] IVIUM files rather than from graphical summaries. The raw 5, 20, 60, and 300 s intermittent-potential datasets were parsed at the hierarchy scan group → cycle → replicate → OCP condition. Repolarization intervals were identified from the transition back to −0.35 V versus Ag/AgCl; expected 1 s sampling was checked within each retained interval. Excess stored charge was calculated from the time-resolved current above the end-of-interval baseline, and cycle-level estimates were summarized first within scan groups and then within experimental replicates so dense time points were never treated as independent replicates. Charge accumulation was fitted with $Q\left(T\right)=Q_{max}\left[1-exp\left(-k_{ch}T\right)\right]$ using the four OCP durations with equal weight across conditions. Positive parameters were estimated in log space. Profile-likelihood 95% intervals used the likelihood-ratio threshold $\chi^{2}\left(1,0.95\right)=3.841459$ [54], and 10,000-resample cluster bootstrap resampled complete replicate summaries within OCP condition. Leave-one-replicate-out fits were used as a leverage sensitivity analysis. Early discharge kinetics were summarized by exponential fits to peak-aligned repolarization current. For independent validation, the median S16 replicate discharge rate was frozen at $k_{rel}=0.35294$ s^−1^ before application to S16b; only trace-specific baseline and amplitude were treated as observation-level nuisance quantities. Nine S16b IVIUM files yielded 150 objectively aligned record-start transients across 20, 60, and 300 s conditions. The source-reported >60% anodic charge-balance fraction was not used as the recovery fraction of the approximately 5 mC transient store and therefore was not used to derive an artificial $k_{eff}$ bound (Supplementary Tables S14 and S15).

The uncertainty framework, therefore, distinguishes three evidence classes. Archived provenance comprises the original aggregate reference results (12.4 mV median full-BEGREM RMSE, 82/99 improvement count, paired $p=6.27\times 10^{-11}$, and median ΔAICc = 2777). Reconstructed evidence comprises the complete 99-curve full/parsimonious predictions, RMSE distribution and bootstrap intervals, paired ΔRMSE vectors, individual AICc values, residual diagnostics, parameter-boundary diagnostics, and full-layer S02 ↔ S03 LOSO. Empirical transient evidence comprises S16 $Q_{m}$ profile likelihood, replicate-cluster uncertainty, leave-one-replicate sensitivity, and independent S16b transfer testing. Only the exact numerical trajectory of the legacy integrated optimizer, including its source-specific residual weights/scales and historical per-curve fit objects, remains irrecoverable; no reconstructed quantity is relabeled as that historical object (Supplementary Tables S12–S22).

## 3. Results

### 3.1. Distinct Electron-Transfer Pathways Produce Resolvable Bioelectrochemical Signatures

The first hypothesis predicted that electron-transfer route and mediator availability would generate experimentally resolvable signatures. Figure 3 organizes source-reported evidence supporting this interpretation. Figure 3A,E provide representative graphical reconstructions of polarization and voltammetric behavior; Figure 3B summarizes mediator-associated changes in power output; Figure 3C contrasts the reported instantaneous and steady-state 2-AQS responses (3.76-fold difference); Figure 3D summarizes the strong mediator-associated impedance contrast. These graphical summaries are used to illustrate mechanistic patterns rather than as additional raw observations. The underlying mediator and impedance contrasts are consistent with the source experiments [21] and established EIS interpretation in microbial electrochemical systems [52]. Figure 3F maps those experimentally distinguishable signatures onto the functional DET/MET classes represented in Equations (6)–(10).

### 3.2. Redox State Constrains Microbial Metabolic Behavior

The second hypothesis evaluated whether redox state provides a measurable constraint on microbial metabolic behavior. Figure 4 now integrates the source-level identifiability analysis with the experimental redox-context evidence. Figure 4A compares leave-one-condition-out prediction errors for the constant, substrate-only, ORP-only, and joint ORP-plus-substrate models; the ORP-only model had the lowest raw RMSE (34.54 mmol mol^−1^), whereas the joint model deteriorated to 68.23 mmol mol^−1^ under cross-condition prediction. Figure 4B shows the near collinearity between ORP and residual glycerol across the four controlled steady states (r = 0.99796, VIF = 244.85, condition number = 31.26). Figure 4C reports the controlled-ORP 1,3-propanediol yield comparison, which increased from 149 to 234 mmol mol^−1^ between −462 and −250 mV (approximately 57%) [26]. Figure 4D summarizes the corresponding source-reported off-gas $O_{2}$ observations, increasing from approximately 4% to 6% [26]. Figure 4E summarizes an independent metabolic perturbation in which production, productivity, and yield increased by approximately 18%, 20%, and 7%, respectively [27]. Figure 4F summarizes the BEGREM interpretation: environmental redox context (ORP, pH, $O_{2}$, and $H_{2}$) constrains the calibrated $f_{ORP}$ response and downstream metabolite formation, while the present four-state design does not permit stable attribution of an independent causal ORP coefficient.

Leave-one-condition-out analysis provided a quantitative but deliberately qualified test of the redox term. Mean normalized RMSE was 1.155 for the constant model, 0.836 for substrate-only response, and 0.783 for ORP-only response. The ORP formulation, therefore, reduced mean prediction error by approximately 6.3% relative to substrate-only kinetics and by 32.2% relative to the constant model. However, ORP and residual substrate were almost perfectly correlated in the controlled dataset (r = 0.998). These data support ORP as a biologically meaningful constraint on metabolism but do not identify a causal ORP contribution independent of substrate depletion. The archived normalized comparison is retained in Supplementary Table S6, while the source-level identifiability diagnostics are reported in Supplementary Table S10.

The source-level collinearity reanalysis made this limitation quantitative. Across the four controlled steady states, ORP and residual glycerol were correlated at r = 0.99796, corresponding to VIF = 244.85; the standardized two-predictor design had a condition number of 31.26. In leave-one-condition-out prediction of 1,3-propanediol yield, raw RMSE was 53.80 mmol mol^−1^ for the constant model, 34.54 mmol mol^−1^ for ORP-only, 40.56 mmol mol^−1^ for substrate-only, and 68.23 mmol mol^−1^ for the joint ORP-plus-substrate model. The deterioration of the joint model under cross-condition prediction is consistent with severe multicollinearity and indicates that the four-state design does not support stable coefficient-specific attribution of ORP and substrate effects. These source-level diagnostics are reported in Supplementary Table S10; the normalized constant/substrate/ORP comparison remains an archived aggregate record in Supplementary Table S6 and is not used to claim independent ORP causality.

### 3.3. Transient Responses Support a Dynamic Reducing-Equivalent Reservoir

The third hypothesis concerned temporal memory in microbial reducing-equivalent availability. Figure 5 now combines the source-level intermittent-potential response with the quantitative raw data identification and external validation of the effective $Q_{m}$ state. Figure 5A shows representative current-density responses under continuous and 5, 20, 60, and 300 s intermittent-potential regimes [36]. Figure 5B summarizes stored charge across OCP duration, with replicate-level medians of 0.379, 1.933, 1.974, and 3.721 mC at 5, 20, 60, and 300 s, respectively. Figure 5C shows the condition-balanced saturation fit $Q\left(T\right)=Q_{max}\left[1-exp\left(-k_{ch}T\right)\right]$, yielding $Q_{max}=3.575$ mC and $k_{ch}=0.02063$ s^−1^. Figure 5D reports the corresponding profile-likelihood 95% intervals ($Q_{max}$: 2.302–5.023 mC; $k_{ch}$: 0.00634–0.10633 s^−1^). Figure 5E summarizes independent S16b transfer testing of the frozen S16-derived discharge rate: median RMSE = 0.02524 A m^−2^, median MAE = 0.02228 A m^−2^, median $R^{2}=-0.877$, and 38/150 traces with $R^{2}>0$. Figure 5F states the effective-state interpretation used by BEGREM: $Q_{m}$ captures history-dependent, kinetically accessible reducing equivalents, while the limited S16-to-S16b transfer demonstrates that discharge-rate parameters remain system-specific.

A memoryless mapping I=f(S,X,E) cannot reproduce a history-dependent current excess after an open-circuit interval when the imposed electrode potential returns to the same value, whereas Equation (5) retains that history through $Q_{m}$. Polarization-only fitting nevertheless indicated weak practical identifiability of recharge-related degrees of freedom: recharge-related solutions were boundary-sensitive or non-unique in quasi-steady polarization fits. Supplementary Table S4 specifies $Q_{m}\left(0\right)$ as non-negative without imposing a finite upper bound. The polarization analysis, therefore, uses a parsimonious $Q_{m}$-depletion representation when recharge cannot be identified, while recharge/discharge identification is reserved for dedicated transient OCP-repolarization data.

This distinction is central to the interpretation of $Q_{m}$. Quasi-steady polarization remains structurally insufficient to separate all $Q_{m}$-related electrochemical parameters, whereas OCP-repolarization supplies the temporal excitation required for practical recharge/discharge identification. Parameter reliability and state necessity are therefore treated as related but distinct questions: the static sensitivity analysis addresses structural separability, and the raw transient analysis addresses practical kinetic estimation.

Across the four OCP durations, replicate-level stored-charge summaries increased from a median 0.379 mC at 5 s to 1.933 mC at 20 s, 1.974 mC at 60 s, and 3.721 mC at 300 s. A condition-balanced saturation fit gave $Q_{max}=3.575$ mC and $k_{ch}=0.02063$ s^−1^ (charging time constant 48.5 s), with a condition-median RMSE of 0.466 mC. Profile-likelihood 95% intervals were 2.302–5.023 mC for $Q_{max}$ and 0.00634–0.10633 s^−1^ for $k_{ch}$. Leave-one-replicate-out sensitivity gave $Q_{max}$ = 3.007–3.847 mC and $k_{ch}$ = 0.01245–0.09207 s^−1^. The condition-stratified replicate bootstrap was much wider and reached the upper search/domain scale for $Q_{max}$ in some resamples, reflecting the strong leverage of heterogeneous 300 s replicates. It is therefore reported as a robustness diagnostic rather than as a precise population interval. The finite profile interval is interpreted as model-conditional practical identifiability (Supplementary Table S14).

The static structural-sensitivity screen remains consistent with the transient results. For the DET submodel, normalized sensitivities of $Q_{m}$ and $K_{Q}$ are exactly opposing within the static saturation term, while $j_{0,D}$ supplies a confounded multiplicative direction; the screening matrix remains rank 2 of 4 with condition number approximately 3.25 × 10^17^. In contrast, the raw repolarization traces provide a directly time-resolved discharge observable. The median S16 replicate discharge estimate was $k_{rel}=0.35294$ s^−1^. When this rate was frozen and applied without structural refitting to 150 objectively aligned S16b transients, median RMSE was 0.02524 A m^−2^ (hierarchical-bootstrap 95% interval 0.01251–0.05176), and median MAE was 0.02228 A m^−2^(0.01176–0.04169). Median $R^{2}$ was −0.877, and only 38/150 transients (25.3%) had $R^{2}>0$; median integrated-charge error was −0.284 C m^−2^ (bootstrap interval −0.410 to −0.088 C m^−2^). Thus, the independent dataset does not support universal transfer of a single S16 discharge-rate constant. Instead, it shows that $Q_{m}$ state necessity and parameter portability are separate questions: the transient-memory architecture is mechanistically motivated, but kinetic rates require system-specific calibration and uncertainty propagation (Supplementary Table S15).

### 3.4. Transport Generates Spatially Heterogeneous Redox Microenvironments

The fourth hypothesis tested whether bulk-state representations are sufficient when transport generates local physicochemical gradients. Figure 6 separates the spatial argument into six panels. Figure 6A depicts the BEGREM−1D domain from lumen through mucus and the microbial zone toward the host interface, with convection, diffusion, and reaction along the spatial coordinate. Figure 6B–E show the representative directions of pH, ORP, and electric-potential gradients constrained by microsensor measurements [37]; these are mechanistic reconstructions of published gradient behavior rather than newly measured human-colon profiles. Figure 6F links transport to temporal product emergence under forced flow, with acetate observed on the order of 10 days and n-caproate on the order of 45 days [33]. Together, the panels support the use of Equation (13) when positional data are available.

Forced-flow experiments additionally showed that hydrodynamic intervention removed locally elevated cathodic pH, redistributed $H_{2}$, and reduced the reported system-level energy demand from 221 to 136 kWh kg^−1^ $H_{2}$ [33]. For BEGREM, the mechanistically relevant result is the change in chemical boundary conditions rather than the engineering energy metric itself. A zero-dimensional model can reproduce only a bulk state and therefore cannot, by construction, reproduce $C_{i}\left(x,t\right)$ gradients; the one-dimensional extension is retained specifically for applications in which spatial measurements justify the added complexity.

### 3.5. BEGREM Predictive Reconstruction Recovers Within-Curve Performance but Reveals Study-Level Transfer Penalties

The predictive evaluation separates archived provenance from independently reconstructed curve-level evidence. The archived comparison reported median held-out RMSEs of 31.1, 73.1, 26.8, 185.0, 12.4, and 13.8 mV for Ohmic, quadratic, Tafel–Ohmic, AOC, full BEGREM, and parsimonious BEGREM, respectively, with full BEGREM improving 82/99 eligible curves. BEGREM MODEL complete independently recovered the same predictive scale from preserved point-level data: reconstructed medians were 31.07, 73.03, 26.90, 185.39, 12.147, and 13.256 mV, respectively. Figure 7A displays the archived and reconstructed model-error scales side by side, demonstrating that the reconstruction reproduces the historical error magnitude without using the archived targets during fitting.

Across the 99 reconstructed full-BEGREM curves, the held-out RMSE median was 12.147 mV with a 20,000-resample complete-curve bootstrap 95% interval of 10.406–14.736 mV and a Book_ID cluster-bootstrap sensitivity interval of 10.440–14.338 mV (Figure 7B). The RMSE IQR was 8.264–20.324 mV and the median held-out MAE was 10.443 mV. The median remained between 12.129 and 12.400 mV after deleting any single curve and between 12.129 and 12.654 mV after deleting any recoverable Book_ID cluster, indicating that the reconstructed central estimate is not driven by one trajectory or recoverable cluster.

The primary paired predictive audit used only classical forms whose equations are recoverable without legacy ambiguity. The curve-specific comparator min (Ohmic, quadratic) had a median held-out RMSE of 25.823 mV. The paired ΔRMSE (classical comparator minus full BEGREM) had a median of 7.700 mV with a 95% complete-curve bootstrap interval of 4.695–18.175 mV; reconstructed full BEGREM had lower RMSE in 81/99 curves, and the one-sided paired Wilcoxon test gave $p=7.20\times 10^{-11}$ (Figure 7C). The Book_ID cluster-bootstrap interval for the median paired ΔRMSE was 4.695–17.713 mV. A secondary sensitivity that also included reconstructed Tafel-like and canonical AOC forms yielded a median paired ΔRMSE of 1.852 mV (95% interval 0.367–3.160 mV), 63/99 RMSE wins, and p=0.00547. Because the exact legacy Tafel–Ohmic/AOC curve-level parameterizations were not retained, this four-model result is reported only as sensitivity and does not replace the archived 82/99 comparison.

Individual AICc values are available for all 99 reconstructed curves. In the primary exact-reconstructable audit, the median ΔAICc (best of Ohmic/quadratic minus full BEGREM) was 1872.5 with IQR 993.1–2796.3, and full BEGREM had lower AICc in 95/99 curves (Figure 7D). The four-model sensitivity gave 92/99 AICc wins with the same median to displayed precision but a narrower IQR of 992.5–2632.4. These large point-wise contrasts are numerically compatible with dense trajectories, but their interpretation is deliberately conservative: the median lag^−1^ correlation of held-out full-BEGREM residuals was 0.996 (IQR 0.980–0.998), demonstrating strong serial dependence among adjacent polarization points. AICc is therefore retained as a secondary within-curve complexity criterion rather than an effect size or standalone evidence of external generalizability.

The full and parsimonious reconstructed variants differed less consistently out of sample than their aggregate medians suggest. Full BEGREM had median RMSE 12.147 mV compared with 13.256 mV for the parsimonious depletion form, but the paired median parsimonious-minus-full difference was effectively zero; full BEGREM was lower in 61/99 curves, and the one-sided paired hold-out comparison was not significant (p=0.243; Figure 7E). Training AICc favored the full variant in 61/99 curves (median $\Delta {AICc}_{pars-full}=445.0$; $p=7.15\times 10^{-10}$), indicating that additional recharge flexibility improves training likelihood without a uniform forward-prediction gain. The reconstructed parameter pattern is consistent with weak recharge identifiability in quasi-steady polarization: $Q_{m,0}$ was >0.999 in 81.8% of curves, and $k_{r}$ was <10^−6^ in 33.3%.

Study-level transfer was substantially more difficult than within-curve extrapolation. In the reconstructed five-parameter full-layer LOSO, training on S03 and holding out S02 produced a median complete-curve RMSE of 75.94 mV (95% curve bootstrap 66.01–79.11 mV), whereas training on S02 and holding out S03 produced 104.15 mV (84.31–129.14 mV; Figure 7F). These fold medians are approximately 6.25-fold and 8.57-fold larger than the reconstructed 12.147-mV within-curve median. The lower-order common-shape LOSO gave the same qualitative conclusion (52.62 and 103.84 mV; Supplementary Table S16). Thus, both external tests identify reactor architecture and operating scale as major transfer modifiers and prevent within-curve accuracy from being interpreted as architecture-independent generalizability.

Table 3 consolidates the archived comparison and reconstructed predictive results. The archived full-BEGREM row preserves the original 12.4-mV, 82/99, and ΔAICc = 2777 provenance. The BEGREM MODEL complete row provides the independently executable 99-curve reconstruction, including its bootstrap interval, primary paired exact-reconstructable audit, and individual AICc summary. The parsimonious reconstruction is retained as the structurally simpler option for quasi-steady sweeps because recharge-related degrees of freedom remain boundary-sensitive without dedicated transient excitation.

Taken together, the within-curve reconstruction and study-held-out tests clarify the distinction between numerical reproducibility and external transferability. The principal within-curve error scale is independently reproducible from preserved raw data, and complete RMSE, residual, paired-effect, AICc, and parameter objects are supplied. At the same time, the much larger study-held-out errors show that the integrated polarization layer is not yet transferable across reactor architectures without harmonized calibration. The only unrecovered element is the exact legacy optimizer/weighting trajectory that generated the archived aggregate objects; it is retained as provenance rather than inferred from the reconstructed results (Supplementary Tables S17–S22).

## 4. Discussion

### 4.1. BEGREM Reframes Bioelectrochemical Signals as Observables of Colonic Microbial Metabolism

BEGREM’s primary contribution is an observation-centered mechanistic layer that connects hidden microbial metabolic-redox states to experimentally accessible electrochemical signals. This places the framework alongside, rather than in competition with, contemporary gut-metabolism models. Genome-scale reconstructions for hundreds of gut organisms established a systematic basis for species-resolved metabolic inference [55], and the AGORA2 expansion to 7302 human-associated microbial strains substantially increased the biochemical scope available for personalized predictions [56]. Individual-based platforms such as BacArena add spatially explicit ecological interactions to metabolic networks [57], whereas SteadyCom imposes community stability while estimating relative microbial abundances [58]. COMETS extends dynamic flux-balance concepts into time and space [59], and broader reviews emphasize that community metabolic modeling must bridge biochemical mechanism with ecological organization [60]. Most recently, personalized community-scale models have predicted individual short-chain-fatty-acid production profiles and linked them to human phenotypes [61]. BEGREM complements these approaches by adding redox, EET, transient reducing-equivalent storage, and electrochemical observability as dynamical constraints that can be measured independently of stoichiometric flux predictions.

The biological plausibility of this observation layer rests on experimentally established EET rather than on extrapolation from power-oriented MFC performance. *F. prausnitzii* uses extracellular shuttles [10,11,12], flavin-based EET occurs in diverse gram-positive organisms [13], and mammalian-gut measurements indicate that extracellular electron exchange can occur in vivo [14]. Accordingly, DET and MET in BEGREM should be interpreted as functional electron-disposal routes whose relative contribution depends on taxonomic composition, microenvironment, and available acceptors; the model does not assume that every colonic taxon contacts or respires an electrode.

The interpretation separates electrochemical observability from biological quantity. A current response can be informative about electron-transfer activity under controlled conditions, but its magnitude is jointly determined by microbial metabolism, redox-carrier availability, the electrode–biofilm interface, abiotic electrocatalysis, and transport. Improved electrical prediction is therefore evidence for the BEGREM observation layer, not direct evidence that SCFA production, community composition, or host physiology is predicted with the same accuracy.

### 4.2. Redox State Is Mechanistically Relevant but Independently Confounded in the Available Design

Controlled-ORP experiments demonstrate that microbial carbon and electron allocation changes across redox states [26], supporting ORP as a mechanistically relevant state variable in Equation (1). The identifiability analysis does not weaken that conclusion; it defines its boundary. Because ORP and residual substrate covary almost perfectly in the available four-condition experiment, the data support redox-sensitive metabolism but do not identify an independent causal coefficient for ORP apart from substrate depletion, pH, and oxygen. The appropriate interpretation is therefore mechanistic relevance with unresolved independent attribution, not absence of a redox effect.

The lower ORP-only leave-one-condition-out error is retained as evidence that ORP contains predictive information about the metabolic state represented in this dataset. It is not used as proof that ORP acts independently of substrate. With r = 0.998 between ORP and residual substrate, a joint ORP-plus-substrate parameterization is severely confounded by the experimental design. Independent causal attribution requires an orthogonal experiment in which imposed redox state is varied while residual substrate is held approximately constant, and vice versa.

The quantitative diagnostics make this boundary explicit. A VIF of 244.85 and condition number of 31.26 show that ORP and residual substrate occupy nearly the same predictor direction, while the joint ORP-plus-substrate model deteriorated to a leave-one-condition-out RMSE of 68.23 mmol mol^−1^ compared with 34.54 and 40.56 mmol mol^−1^ for the ORP-only and substrate-only models. Thus, the present data positively support redox sensitivity but cannot resolve how much of the observed response should be assigned uniquely to ORP. This distinction preserves the biological meaning of redox state while preventing an unsupported causal claim.

### 4.3. Q_m_ Captures Experimentally Observed Redox Memory

The most distinctive BEGREM state, $Q_{m}$, is supported at the level that matters most for the present model: a history-dependent state is required to represent reducing-equivalent accumulation and subsequent release. Intermittent-potential experiments show recoverable charge accumulation during open-circuit intervals and a transient current pulse after repolarization [36]. A memoryless mapping based only on instantaneous substrate, biomass, and electrode state cannot encode that prior open-circuit history. $Q_{m}$ provides a conservation-compatible state, expressed in coulombs, that carries this temporal information between reducing-equivalent generation and DET/MET outflow. Its interpretation as an effective state is deliberate: $Q_{m}$ may integrate reduced cofactors, membrane redox components, extracellular matrix charge, soluble mediators, and other kinetically accessible reducing equivalents without asserting that any one of those pools is the unique molecular carrier.

$Q_{m}$ can be evaluated at two distinct levels: state necessity and parameter identification. The archived BEGREM comparison and reconstructed polarization analyses are consistent with a useful history-dependent latent state, whereas the raw intermittent-potential experiment provides direct temporal excitation for estimating charge accumulation and release. The condition-balanced estimate $Q_{max}=3.575$ mC is of the same order as the source-reported approximately 5 mC transient accumulation, and finite profile-likelihood intervals for $Q_{max}$ and $k_{ch}$ show that the 5–300 s perturbation series contains substantially more charging-kinetic information than quasi-steady polarization. The wider replicate-cluster bootstrap nevertheless demonstrates that model-conditional identifiability does not imply narrow between-reactor uncertainty.

The structural-sensitivity audit and raw-trace analysis, therefore, answer different questions and are mutually consistent. Static DET observations cannot separate $Q_{m}$ from $K_{Q}$ and $j_{0,D}$, producing the previously identified rank deficiency. Time-resolved OCP-repolarization excites the latent state directly and supports practical estimation of a charge-accumulation scale and charging rate. Independent S16b traces then provide a deliberately harder parameter-transfer test: a discharge rate frozen from S16 performed poorly on the median S16b transient (median $R^{2}=-0.877$; 25.3% of traces with $R^{2}>0$), despite finite absolute-error estimates. This result strengthens, rather than weakens, the identifiability argument by separating evidence for a history-dependent state from evidence for universal kinetic parameters. $Q_{m}$ is therefore retained as a mechanistic state representation, whereas $k_{rel}$ is treated as system-specific unless independently calibrated.

### 4.4. Spatial Transport Is a Mechanistically Necessary Extension When Local Gradients Are Resolved

Spatial transport is not an optional embellishment when the observation set contains local gradients. The colon is spatially organized, with oxygen, pH, hydrogen, metabolites, and redox potential varying across lumen, mucus, microbial aggregates, and the epithelial interface [8]. Microsensor studies likewise show that local pH, hydrogen, ORP, and electric potential can diverge strongly from bulk values [37], while forced-flow experiments demonstrate that hydrodynamics redistributes these gradients and changes associated metabolic outputs [33]. Because a zero-dimensional model cannot, by construction, reproduce $C_{i}\left(x,t\right)$, the BEGREM-1D extension is mechanistically required whenever positional data are part of the inference problem.

The remaining uncertainty concerns parameter transfer, not the validity of the transport-reaction balance itself. The present profiles establish support for spatially resolved advection–diffusion–reaction behavior; colon-specific use still requires direct calibration of boundary conditions, effective diffusivities, flow/advection, microbial localization, and host-interface constraints. Accordingly, BEGREM-1D is a transferable mechanistic structure whose quantitative physiological deployment depends on calibration rather than a merely illustrative add-on.

### 4.5. Limitations and Experimental Path Toward Colonic Validation

The remaining limitations delimit transferability, parameter attribution, and physiological scope; they do not negate the empirical support for the constituent mechanisms. First, the 22-study evidence base is intentionally heterogeneous and includes non-colonic bioelectrochemical systems used to isolate mechanisms that are difficult to perturb independently in the gut. Second, the parsed microbiome evidence does not yet provide a complete abundance matrix for community-diversity inference. Third, the spatial curves in Figure 6 are mechanistic representations constrained by published gradients rather than direct human-colon profiles. Fourth, strong ORP-substrate collinearity prevents clean attribution of an independent redox effect. Fifth, $Q_{m}$ is an effective latent state whose molecular composition remains unresolved. These boundaries identify the experiments required for parameter transfer and physiological validation while preserving the module-level mechanistic evidence already demonstrated.

Sixth, the 70/30 high-current split is a within-curve extrapolation test and is not treated as external validation. Both the common-shape and reconstructed full-layer S02 ↔ S03 LOSO analyses yield substantially larger errors, establishing reactor architecture and operating scale as important transfer modifiers. Seventh, the exact historical full-BEGREM optimizer trajectory, source-specific endpoint weights/scales, and legacy per-curve fit objects were not retained. This prevents byte-for-byte recovery of the historical likelihood, but it no longer prevents an auditable curve-level reanalysis: BEGREM MODEL complete supplies 99 reconstructed RMSE distributions, complete paired ΔRMSE vectors, individual AICc values, residual diagnostics, parameter-boundary diagnostics, and a five-parameter study-held-out transfer test. Eighth, $Q_{m}$ charging kinetics are identifiable under dedicated OCP-repolarization excitation, but wide replicate uncertainty and poor S16-to-S16b portability of a frozen $k_{rel}$ require system-specific calibration and uncertainty propagation. Curve-set statistics are therefore interpreted descriptively rather than as population-level biological inference.

The next validation stage should use a gut-mimetic platform that can perturb metabolism, redox, transport, and electron-transfer observables independently. Early gut-on-chip work established simultaneous microbial colonization, fluid flow, and peristalsis-like mechanical cues [62], and modern organ-on-chip frameworks explicitly integrate host-microbiome interactions [63]. Electrochemical gut-on-chip concepts further identify mucosal redox biology as a tractable measurement target [64]. Earlier colon-oriented MFC studies demonstrated that intestinal pH, dissolved oxygen, ORP, and continuous flow can be reproduced experimentally [65,66], while MFC-biosensor research provides a broader basis for treating current as an analytical signal rather than an energetic endpoint [67]. A physiologically meaningful implementation must additionally respect mucus-barrier biology [68], gastrointestinal redox signaling [69], and the steep oxygen landscape of the intestinal mucosa [70]. A decisive experiment would therefore combine an anaerobic luminal microbial compartment, mucus/epithelial boundary conditions, controlled substrate pulses, independently imposed redox perturbations, OCP-repolarization sequences, flow modulation, ORP/pH/$H_{2}$ sensing, metabolite quantification, and a minimally perturbative bioelectrochemical sensing interface.

### 4.6. Independent Evidence Reinforces Mechanistic Interpretation and Defines External Validity

The effective-state interpretation of $Q_{m}$ is consistent with independent literature on charge storage in electroactive biofilms. Electron storage has been reviewed as arising from reduced redox-active electron-transport components and longer-lived storage pools [71], while periodic polarization experiments demonstrated open-circuit charge accumulation and release after repolarization together with increased redox-carrier concentration [72]. Earlier Geobacter experiments recovered stored electrons as a transient current after electrode reconnection and quantitatively modeled charge accumulation and transfer [73], and open-circuit potentiometry subsequently resolved multiple redox reservoirs between intracellular reducing equivalents and the electrode [74]. More recent work further shows that current transients can contain contributions from intracellular carbon-storage metabolism under substrate limitation [75]. Together, these studies support $Q_{m}$ as an effective history-dependent inventory; they do not identify $Q_{m}$ as a single molecular carrier.

The reliability of a latent state must also be separated from identifiability of every parameter that controls it. Contemporary identifiability literature distinguishes structural necessity from practical parameter identifiability and emphasizes that partially observed dynamical models may predict an observable while individual parameter combinations remain weakly constrained [76]. Profile-likelihood workflows provide a route to propagate parameter uncertainty into prediction intervals and to identify parameter combinations that require additional experimental excitation [77]. For BEGREM, this supports the parsimonious depletion-state treatment for quasi-steady polarization data and identifies OCP-repolarization experiments as the appropriate design for estimating recharge kinetics.

Physiological anchoring for the redox environment is provided by in-human ingestible-sensor measurements in healthy participants, which recorded high-temporal-resolution gastrointestinal redox profiles and confirmed a strongly reducing large-intestinal environment [78]. At the same time, ex vivo fecal ORP measurements can be unstable and have failed to discriminate disease status reliably in some settings [79]. These observations reinforce the interpretation of ORP as a context-dependent physicochemical state variable and boundary condition rather than a universal standalone biomarker.

The DET/MET distinction is likewise interpreted functionally. Electrochemical perturbation studies in Shewanella have distinguished flavin-dependent and flavin-independent electron-transfer routes using flavin addition/removal, cell-removal controls, mutants, and voltammetric signatures [80]. Microbial electrochemical sensing reviews similarly emphasize that current is an analytical signal generated by an electrode-microbe system whose response depends on operational and biological conditions [81]. Thus, BEGREM uses DET and MET as mechanistic flux classes and current as a composite observation, not as a direct measure of total gut metabolism.

The evidence terminology follows the statistical principle that dependence structure must be respected when predictive models are evaluated. Random point-level splits can underestimate error when measurements are clustered or repeated [82]. Accordingly, the 70/30 split is retained only as within-curve forward extrapolation. The common-shape S02 ↔ S03 LOSO produced median curve RMSEs of 52.62 and 103.84 mV, and the reconstructed full-layer LOSO produced 75.94 and 104.15 mV. Both are appreciably larger than within-curve summaries. These study-held-out results quantify an architecture-transfer penalty and identify harmonized reactor-specific calibration as a prerequisite for integrated BEGREM transfer; they do not support a claim of broad cross-study generalizability.

The completed recoverable data analysis uses all numerical information supported by the public raw archive. S16 potentiostat traces provide empirical $Q_{m}$ fitting, profile-likelihood intervals, replicate-cluster uncertainty, leave-one-replicate sensitivity, and independent S16b parameter-transfer testing. S02 and S03 provide two independent study-held-out polarization analyses. The BEGREM MODEL complete reconstruction additionally supplies (i) complete-curve and cluster-bootstrap uncertainty for full-BEGREM RMSE, (ii) the 99-value per-curve full/parsimonious RMSE distributions and point predictions, (iii) complete paired ΔRMSE vectors for the exact-reconstructable comparator audit and four-model sensitivity, (iv) individual curve-wise AICc and residual-dependence diagnostics, and (v) study-held-out testing of the reconstructed five-parameter full polarization layer. The exact historical optimizer states and source-specific legacy weights/scales are the only unavailable numerical objects and are not fabricated. All reconstructed objects, scripts, and integrity records are supplied in Supplementary Tables S17–S22 and the BEGREM MODEL complete analysis directory.

Taken together, the evidence supports a bounded interpretation of BEGREM. The existence of temporal redox memory is supported independently of universal parameter portability; the reconstructed within-curve predictive scale is reproducible independently of the archived aggregate targets; and study-held-out transfer is materially weaker than within-curve prediction. The principal remaining uncertainties, therefore, concern parameter transfer across reactor architectures, molecular interpretation of the effective $Q_{m}$ pool, and colon-specific calibration rather than the numerical reproducibility of the within-curve observation layer.

## 5. Conclusions

BEGREM provides an integrated, quantitatively testable architecture for gut-relevant microbial metabolic-redox dynamics by coupling substrate conversion, metabolite production, redox state, direct and mediated EET, transient reducing-equivalent storage, and spatial transport while separating latent biological states from bioelectrochemical observations. The archived within-curve comparison reported full-BEGREM median RMSE = 12.4 mV and improvement in 82/99 curves. Independent BEGREM MODEL complete reconstruction from preserved S02 point-level data reproduced that predictive scale at 12.147 mV (95% complete-curve bootstrap 10.406–14.736 mV), generated all 99 curve-level RMSE/AICc objects, and yielded 81/99 improvements against the exact-reconstructable Ohmic/Quadratic comparator set with median paired ΔRMSE = 7.700 mV. The primary individual AICc audit favored full BEGREM in 95/99 curves, while strong serial residual dependence limits AICc to a secondary within-curve criterion. Raw OCP-repolarization data identify $Q_{max}=3.575$ mC and $k_{ch}=0.02063$ s^−1^ with finite profile-likelihood intervals, whereas independent S16b testing demonstrates limited portability of a single frozen discharge constant. Reconstructed full-layer study holdout yields median RMSEs of 75.94 and 104.15 mV, establishing a substantial cross-architecture transfer penalty. Controlled-redox data support redox-sensitive metabolism while retaining the ORP-substrate identifiability boundary, and published local gradients support the spatial transport extension. The resulting evidence supports mechanistic and within-curve predictive validity while leaving universal parameter transfer and colon-specific physiological validation as explicit experimental requirements.

## Figures and Tables

**Figure 1:**
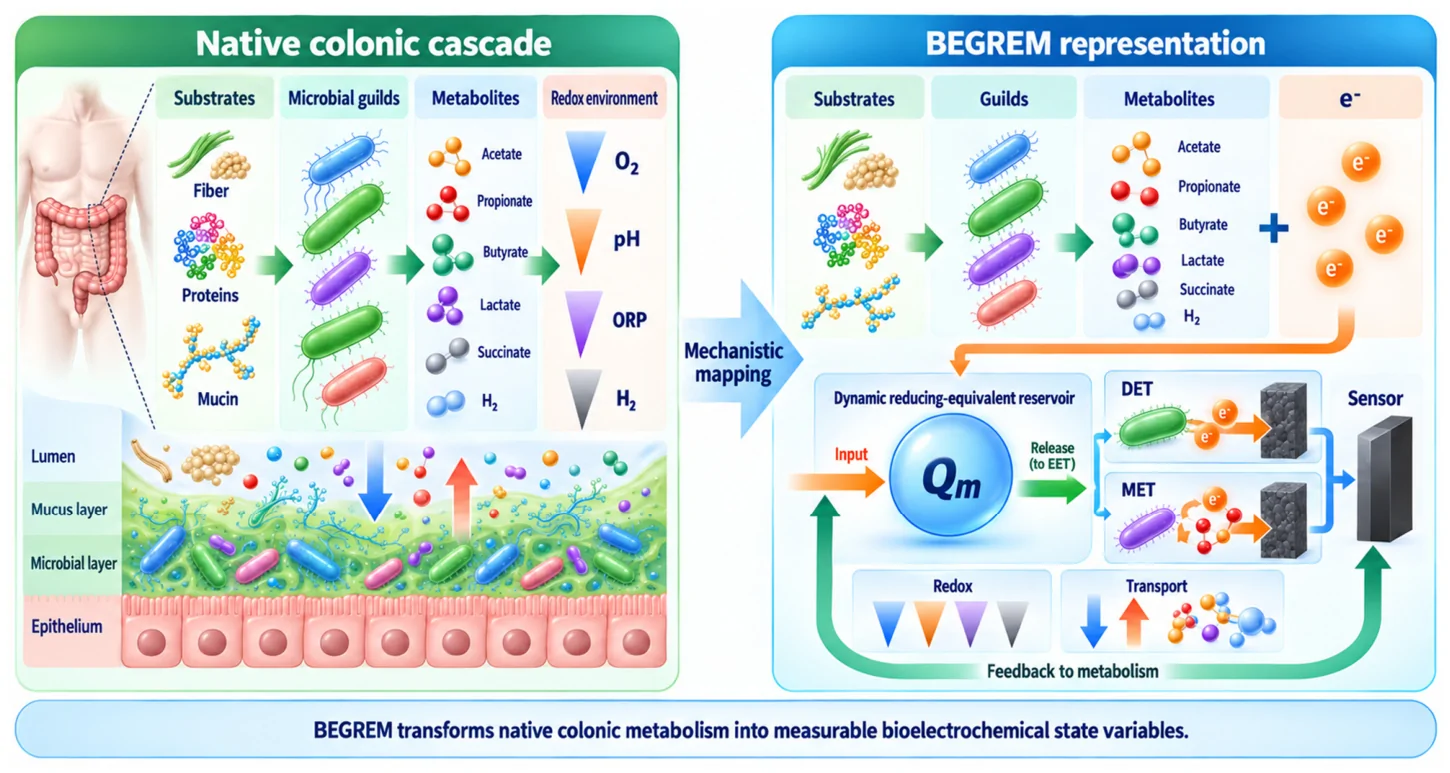
BEGREM conceptual mapping of the native colonic metabolic cascade. The native colonic cascade (**left**) links fermentable substrates, microbial functional guilds, metabolites, and the local redox environment across the lumen–mucus–epithelium axis. The BEGREM representation (**right**) maps these processes to measurable and latent model states, including extracellular electron flow, the dynamic reducing-equivalent reservoir $Q_{m}$, direct extracellular electron transfer (DET), mediated extracellular electron transfer (MET), redox feedback, and transport. The electrode is represented as a bioelectrochemical-sensing interface rather than as the biological endpoint of the model. Created with BioRender.com.

**Figure 2:**
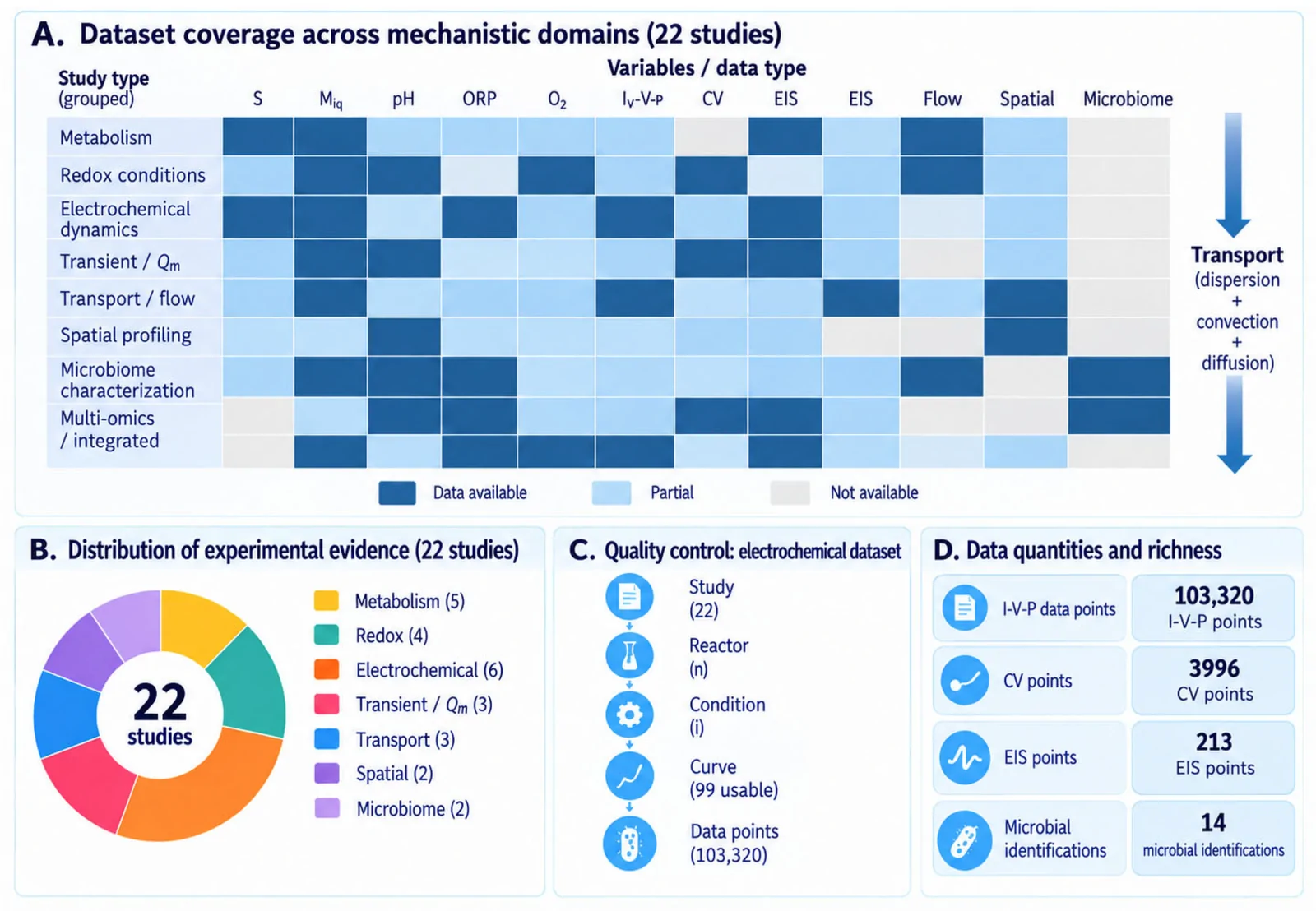
Experimental evidence base and data sufficiency for BEGREM. (**A**) Dataset coverage across mechanistic domains, indicating complete, partial, or unavailable information for metabolism, redox, electrochemical dynamics, transient $Q_{m}$ behavior, flow/transport, spatial profiling, microbiome characterization, and integrated evidence. (**B**) Non-exclusive evidence-category assignments across the 22 public experimental sources; individual studies can contribute evidence to more than one mechanistic category, so category counts need not sum to 22. (**C**) Quality-control hierarchy preserving study, reactor, condition, curve, and point-level dependence and identifying 99 usable electrochemical curves. (**D**) Quantitative richness of the integrated electrochemical dataset: 103,320 current-voltage-power points, 3996 CV points, 213 EIS points, and 14 microbial identifications.

**Figure 3:**
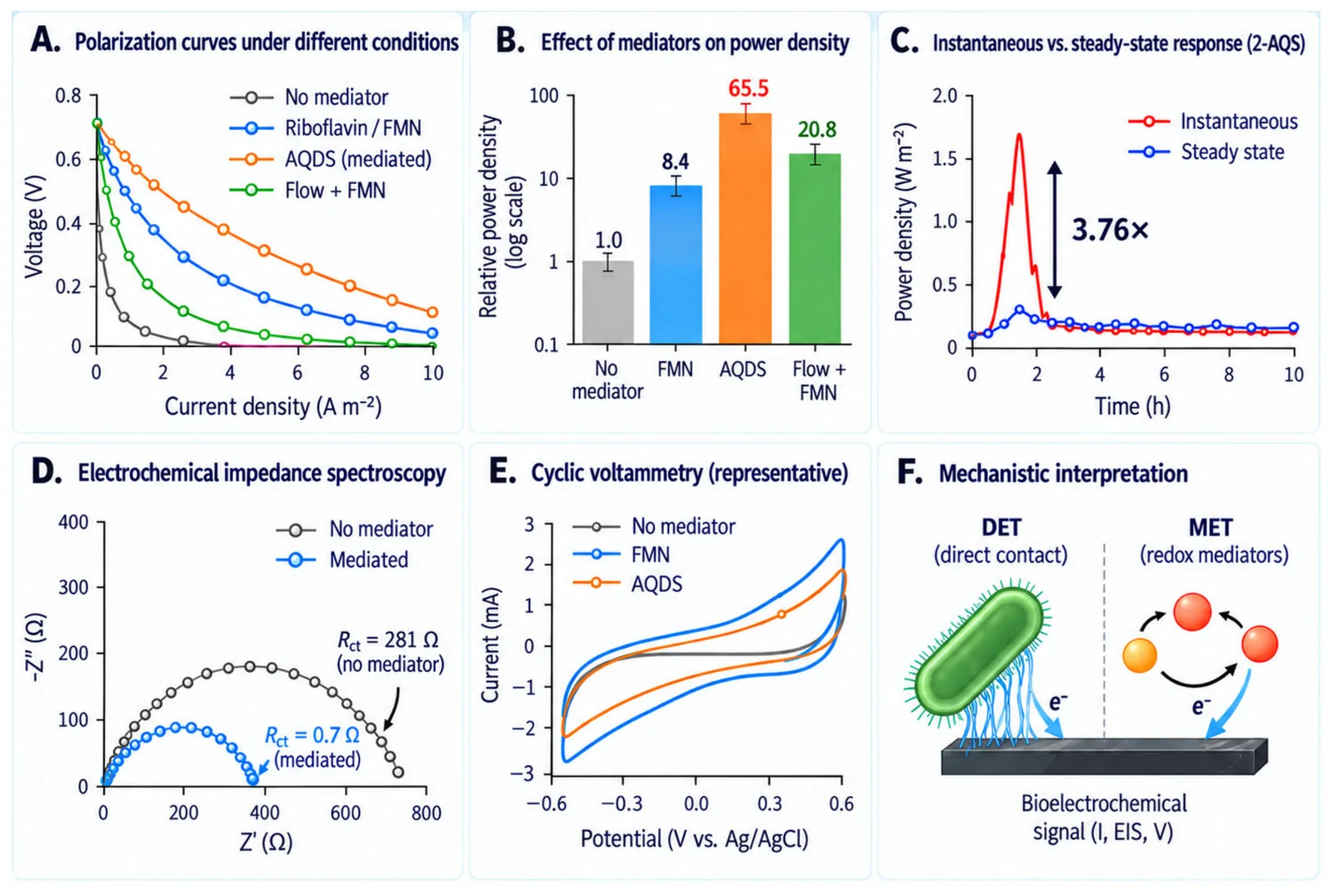
Bioelectrochemical observability of microbial electron-transfer dynamics. (**A**) Representative polarization behavior under different mediator and flow conditions. (**B**) Relative mediator-associated change in power-density output. (**C**) Instantaneous versus steady-state response for 2-AQS, showing a 3.76-fold transient difference. (**D**) Electrochemical-impedance contrast illustrating the mediator-associated decrease in charge-transfer resistance. (**E**) Representative cyclic-voltammetry behavior. (**F**) Mechanistic interpretation separating direct extracellular electron transfer (DET) from mediated extracellular electron transfer (MET). Panels (**A**–**E**) summarize or graphically reconstruct source-reported electrochemical behavior for mechanistic comparison; they are not treated as additional independent raw observations beyond the source data documented in Supplementary Tables S1–S3.

**Figure 4:**
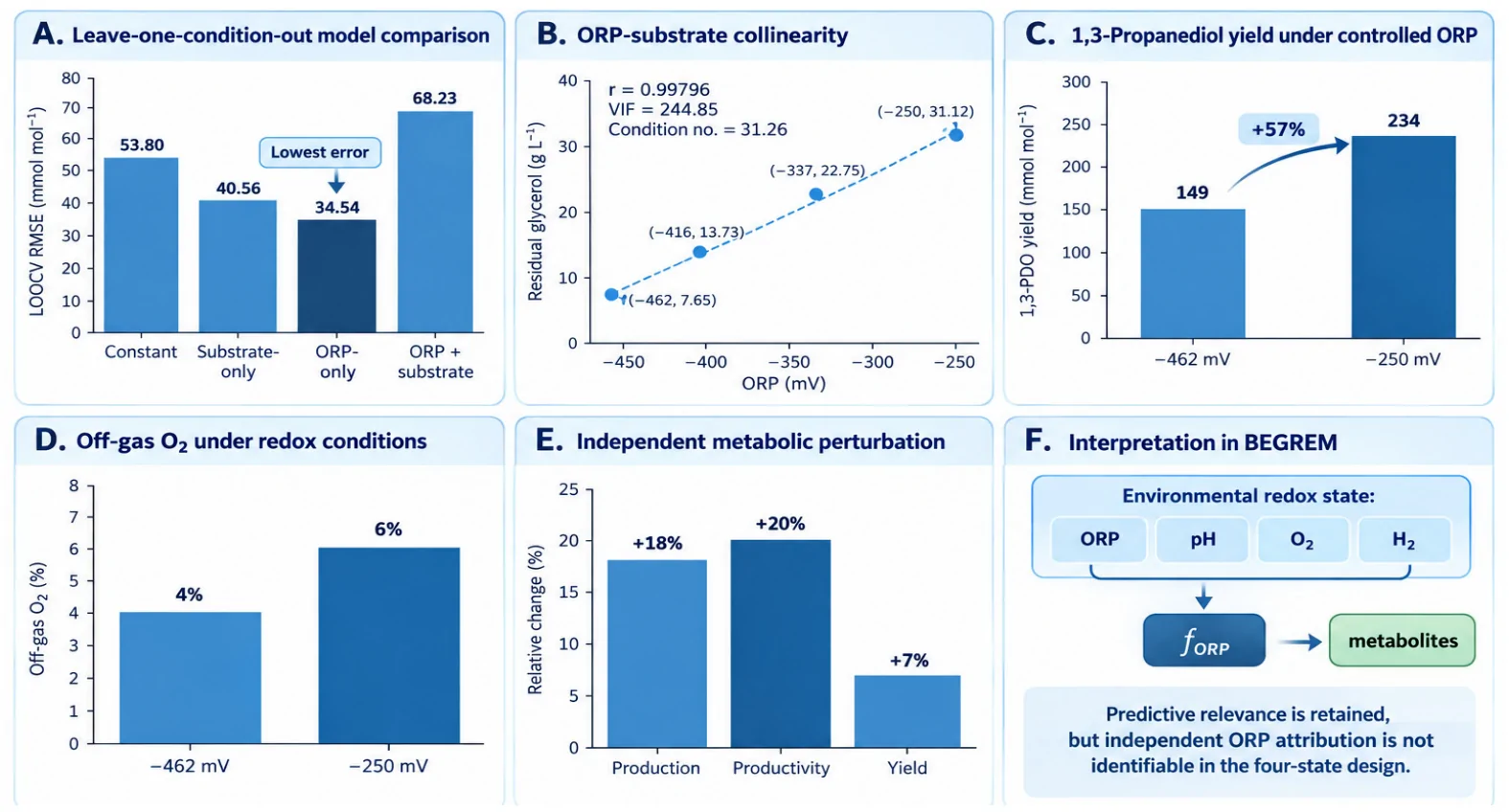
Redox state constrains microbial metabolic behavior. (**A**) Leave-one-condition-out source-level comparison of 1,3-propanediol yield models: constant (RMSE 53.80 mmol mol^−1^), substrate-only (40.56 mmol mol^−1^), ORP-only (34.54 mmol mol^−1^), and joint ORP-plus-substrate (68.23 mmol mol^−1^). (**B**) ORP-residual glycerol collinearity across the four controlled steady states (r = 0.99796; VIF = 244.85; condition number = 31.26), illustrating why independent coefficient attribution is unstable. (**C**) Controlled-ORP comparison of 1,3-propanediol molar yield, increasing from 149 to 234 mmol mol^−1^ between −462 and −250 mV (approximately +57%) [26]. (**D**) Source-reported off-gas $O_{2}$ observations under the same controlled-redox conditions, increasing from approximately 4% to 6% [26]. (**E**) Independent metabolic perturbation showing approximately +18% production, +20% productivity, and +7% yield [27]. (**F**) BEGREM interpretation linking environmental redox state (ORP, pH, $O_{2}$, and $H_{2}$) to the calibrated $f_{ORP}$ modifier and metabolite generation. The figure supports redox relevance but does not imply that ORP and substrate effects are independently identifiable in the available four-state design; source-level diagnostics are reported in Supplementary Table S10.

**Figure 5:**
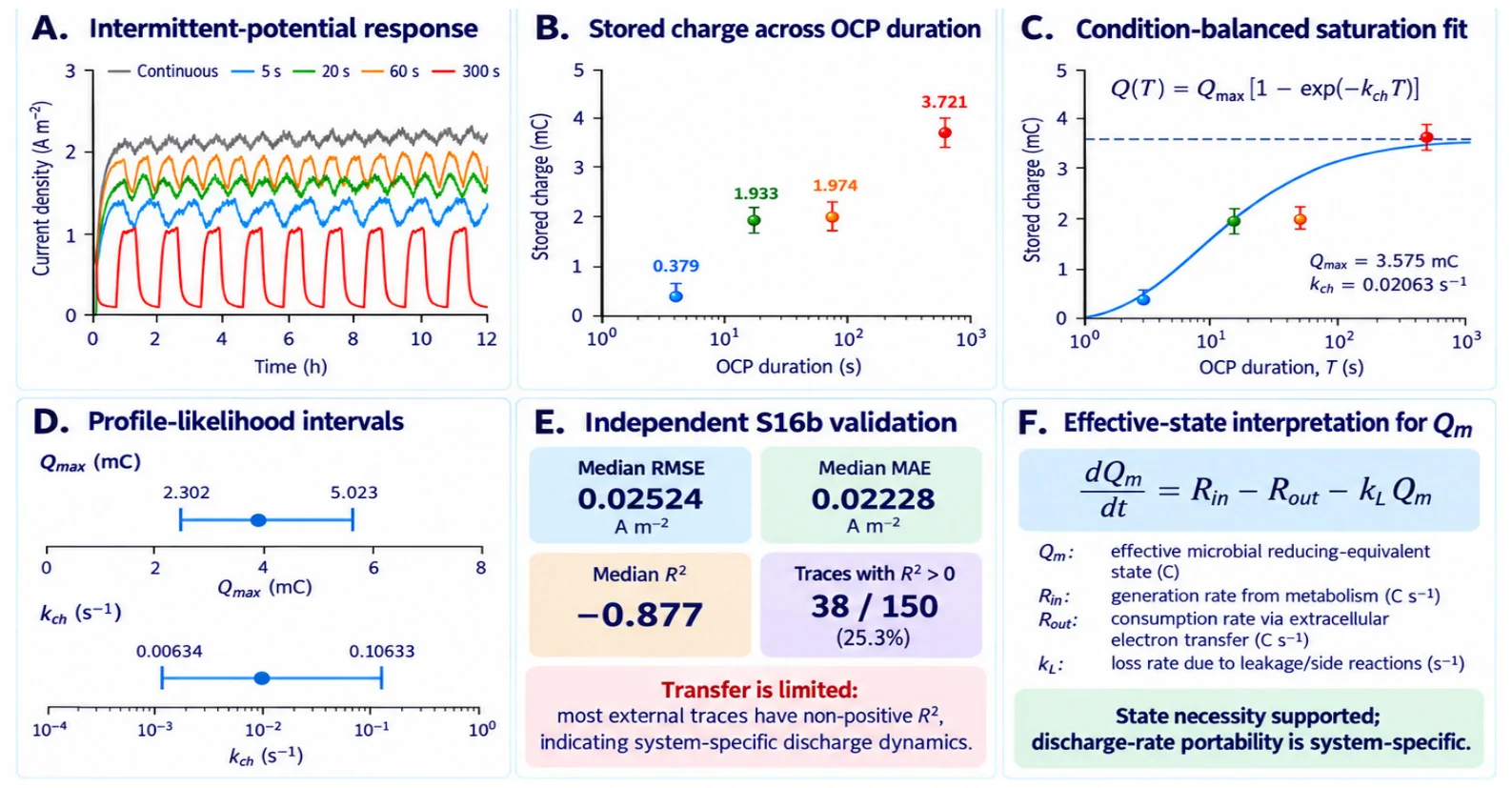
Dynamic microbial-reducing equivalent reservoir ($Q_{m}$): empirical identification and external validation. (**A**) Representative current-density response under continuous and intermittent-potential regimes (5, 20, 60, and 300 s) from the Pereira et al. experimental context [36]. (**B**) Replicate-level stored-charge summaries across OCP duration: 0.379 mC at 5 s, 1.933 mC at 20 s, 1.974 mC at 60 s, and 3.721 mC at 300 s. (**C**) Condition-balanced saturation fit, $Q\left(T\right)=Q_{max}\left[1-exp\left(-k_{ch}T\right)\right]$, with $Q_{max}=3.575$ mC and $k_{ch}=0.02063$ s^−1^. (**D**) Profile-likelihood 95% intervals for $Q_{max}$ (2.302–5.023 mC) and $k_{ch}$ (0.00634–0.10633 s^−1^). (**E**) Independent S16b validation of the frozen S16-derived discharge rate: median RMSE 0.02524 A m^−2^, median MAE 0.02228 A m^−2^, median $R^{2}$ − 0.877, and 38/150 traces (25.3%) with $R^{2}>0$, demonstrating limited parameter portability. (**F**) Effective-state interpretation of $Q_{m}$ using $\frac{dQ_{m}}{dt}=R_{in}-R_{out}-k_{L}Q_{m}$. $Q_{m}$ represents a history-dependent inventory of kinetically accessible microbial-reducing equivalents rather than a unique intracellular compartment, the data support state necessity while indicating that discharge-rate calibration is system-specific.

**Figure 6:**
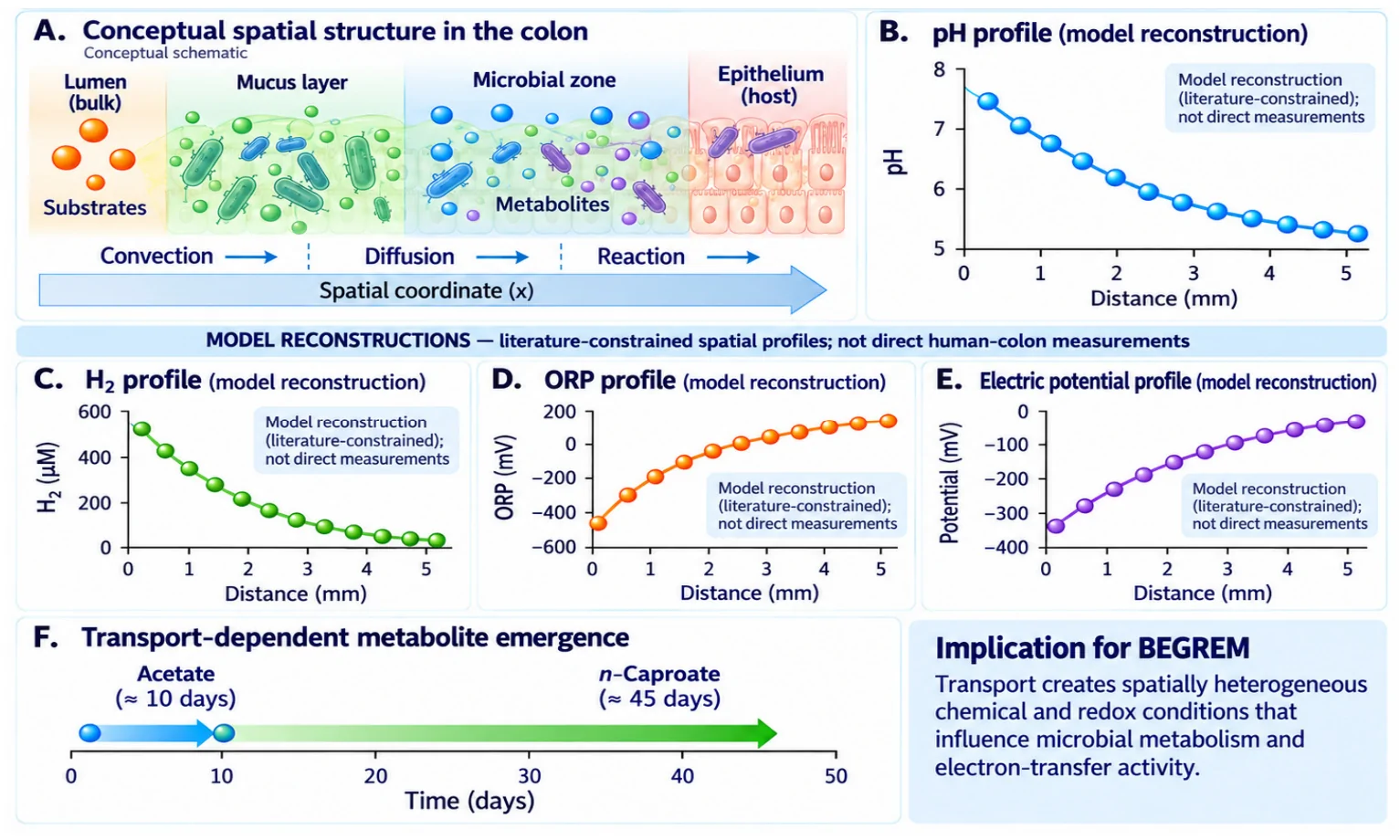
Transport-generated microenvironments constrain microbial redox dynamics. (**A**) Conceptual spatial domain from lumen through mucus and the microbial zone to the host interface, with convection, diffusion, and reaction. (**B**) Representative pH gradient constrained by published microsensor behavior. (**C**) Representative $H_{2}$ profile. (**D**) Representative ORP profile. (**E**) Representative electric-potential profile. Panels (**B**–**E**) encode published bioelectrochemical gradients and are not newly measured human-colon profiles. (**F**) Transport-dependent metabolite emergence, illustrating acetate at approximately 10 days and n-caproate at approximately 45 days under forced-flow conditions. Subfigure (**A**) was created with BioRender.com.

**Figure 7:**
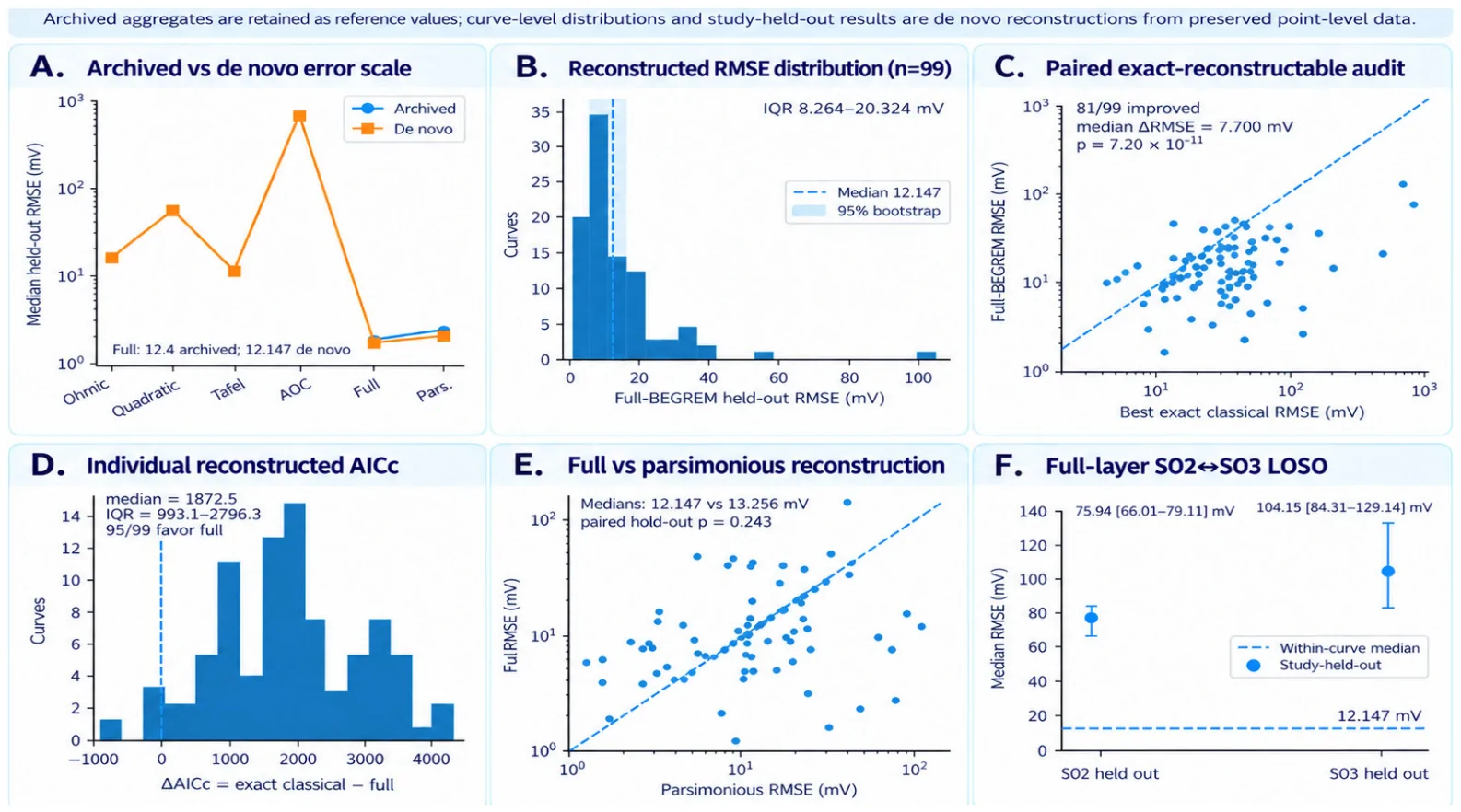
BEGREM MODEL complete reconstructed predictive performance and transferability audit. (**A**) Archived versus de novo median held-out RMSE across Ohmic, quadratic, Tafel–Ohmic/Tafel-like, AOC, full BEGREM, and parsimonious BEGREM; archived aggregates are retained as provenance and were not used as reconstruction targets. (**B**) Distribution of reconstructed full-BEGREM held-out RMSE across 99 eligible curves, with median 12.147 mV and complete-curve bootstrap 95% interval 10.406–14.736 mV. (**C**) Paired exact-reconstructable comparison against min(Ohmic, quadratic): 81/99 curves favor full BEGREM, median ΔRMSE = 7.700 mV, $p=7.20\times 10^{-11}$. (**D**) Individual reconstructed AICc audit against the exact-reconstructable comparator set: median ΔAICc = 1872.5, IQR 993.1–2796.3, with 95/99 curves favoring full BEGREM. (**E**) Paired full-versus-parsimonious held-out comparison, showing similar out-of-sample performance despite greater training flexibility in the full variant. (**F**) Reconstructed five-parameter full-layer S02 ↔ S03 study-held-out validation, with fold median RMSEs of 75.94 and 104.15 mV and the reconstructed within-curve median shown as reference. Curve-level uncertainty is descriptive for the archived/reconstructed curve set and is not interpreted as a biological-population confidence interval.

**Table 1:** Public experimental evidence used to develop, constrain, compare, or provide mechanistic support for BEGREM. The Reference column follows the MDPI numerical citation system; persistent public data identifiers are retained in the supplementary source-data manifest.

Study	Evidence System	Key Variables	BEGREM Role	Reference
Zhang et al., 2024	Redox-mediated flow bioelectrochemistry	I, V, power, EIS, flow, mediators	Calibration	[21]
Amen et al., 2021	Rainwater MFC community	OCP, I-V-P, CV, EIS, microbiome	Within-study polarization comparison	[22]
Serra, 2019	Artificial-wastewater MFC	Longitudinal V/I/P, polarization	Reference comparison	[23]
D’Ugo et al., 2021	Hypoxic hydrocarbon MFC	V, j, power, biofilm, metagenomics	Mechanistic support	[24]
Arbter et al., 2019	Electro-fermentation	ORP, lipids, metabolic model	Redox support/prior	[25]
Arbter et al., 2022	Continuous controlled-ORP BES	ORP, metabolites, fluxes, pH, $O_{2}$	Calibration	[26]
Pateraki et al., 2023	Bioelectrochemical succinate fermentation	Production, productivity, RNA response	Metabolic-redox support	[27]
de Smit et al., 2021	Microbial electrosynthesis	Potentiostat, CV, acids, metals	Electrochemical support	[28]
Zegers et al., 2025	Miniaturized flow-through MES	ECSA, porosity, j, acids	Replicability assessment	[29]
Zegers et al., 2025	MES multivariable modeling	pH, *p*CO_2_, pH_2_, acetate, products	Statistical comparison	[30]
Agudelo-Escobar et al., 2022	Coffee-wastewater BES	COD, current/power, microbiome	Mechanistic/community support	[31]
Chatzipanagiotou et al., 2022	Ni-doped 3D biocathodes	j, charge, pH, products, NGS	Abiotic/biotic support	[32]
de Smit et al., 2023	Forced-flow 3D cathode	Flow, local pH, H_2_, potential, products	Calibration	[33]
de Smit et al., 2023	Abiotic HER catalyst	j, H_2_, overpotential, trace metals	Abiotic control	[34]
de Smit et al., 2024	Catalyst-coupled MES	j, H_2_, microbiome, products	Mechanistic support	[35]
Pereira et al., 2022	Intermittent bioanode	j, $Q_{stored}$, biomass, EPS, CE	Calibration	[36]
de Smit et al., 2023	Microsensor profiling	ORP, pH, H_2_, electric potential	Spatial calibration	[37]
Du et al., 2026	Bidirectional EET	Anodic/cathodic current, potential	Mechanistic support	[38]
Joo et al., 2021	Biofilm spectral impedance	Complex conductivity, frequency, biofilm	EIS mechanistic support	[39]
Georg et al., 2021	BES degradability	COD/VFA, conversion, $NH_{4}$ potential	Conversion support	[40]
Losantos et al., 2021	Ammonium-recovery BES	j, $NH_{4}$ transport, airflow, energy	Transport support	[41]
Tajdid Khajeh et al., 2020	H-type MFC electrode study	V, P, j, EIS, CV	Electrode comparison	[42]

**Table 2:** Principal BEGREM state variables, mechanistic fluxes, and observables. $Q_{m}$ is defined as an effective latent reducing-equivalent state rather than as a literal intracellular compartment.

Symbol	Meaning	Unit	Model Role	Basis
S	Available fermentable substrate	mass or mol volume^−1^	Dynamic state	Canonical substrate kinetics [43,46]
X	Active microbial biomass or functional guild biomass	mass volume^−1^	Dynamic state	Growth/decay and reactor balances [43,44,45,46]
$M_{red}$	Reduced soluble mediator pool	mol volume^−1^	Dynamic state	Mediator mass balance [45,47,49]
*M_ox_*	Oxidized soluble mediator availability	mol volume−1	Exogenous/algebraic input	Source-specific mediator level; $M_{ox}=M_{T}-M_{red}$ when $M_{T}$ is known
*f_pH_*, *f_ORP_*, *f_O2_*	Environmental response modifiers	dimensionless	Exogenous/calibrated modifiers	Bounded in [0,1]; default 1 when no independent modifier is supplied
*η_D_*, *η_M_*	DET and MET overpotentials	V	Algebraic electrochemical inputs	Electrode potential minus pathway equilibrium potential in a common reference frame
$Q_{m}$	Effective microbial reducing-equivalent reservoir	C	Dynamic latent state	BEGREM latent state; transient evidence [36]
$E_{ORP}$	Mixed redox potential of the microbial environment	V	Algebraic/observed state	Nernst and mixed-potential basis [50]
$I_{DET}$	Direct extracellular electron-transfer current	A	Mechanistic flux	Butler–Volmer-type EET [47,51]
$I_{MET}$	Mediator-assisted extracellular electron-transfer current	A	Mechanistic flux	Mediator-assisted EET [45,47,49,51]
$I_{obs}$	Measured current	A	Observation	Observation model with abiotic control [34,48]

**Table 3:** Archived and independently reconstructed within-curve held-out high-current prediction results for the 99 eligible polarization curves. Archived aggregate outputs are retained for provenance. BEGREM MODEL complete rows report de novo curve-level reconstruction from preserved point-level data and are not back-calculated from the archived targets.

Model	Mechanistic Level	Median Within-Curve Held-Out RMSE (mV)	Curve-Level Improvement	Interpretation
Ohmic	Classical electrical	31.1	-	-
Quadratic	Empirical curvature	73.1	-	-
Tafel–Ohmic	Activation + Ohmic	26.8	-	-
AOC	Activation + Ohmic + concentration	185.0	-	-
BEGREM full (archived)	Dynamic latent	12.4	82/99 vs. best classical (archived descriptive count)	Archived median ΔAICc = 2777 (IQR 1933–3891); archived reference result
BEGREM parsimonious (archived)	$Q_{m}$ depletion state	13.8	Historical curve-level vector unavailable	Archived median; exact legacy per-curve objects not retained
BEGREM full–de novo reconstruction	Recovered five-parameter latent polarization layer	12.147 [10.406–14.736]	81/99 vs. exact-reconstructable comparator; 63/99 in four-model sensitivity	Primary exact audit: median ΔRMSE 7.700 mV [4.695–18.175]; median ΔAICc 1872.5 (IQR 993.1–2796.3); 95/99 AICc wins
BEGREM parsimonious—de novo reconstruction	Fixed $q_{0}=1$, *k_r_* = 0 depletion layer	13.256 [11.633–17.027]	Full lower in 61/99; paired hold-out p=0.243	Structurally simpler quasi-steady option; full training AICc advantage does not translate to uniform held-out gain

## Data Availability

All source datasets analyzed in this study are publicly accessible through the article references listed in Table 1 and the persistent repository identifiers compiled in Supplementary Table S1. The Supplementary Material archive contains the original-value BEGREM workbook; point-level I-V-P, CV, EIS, and microbial-identification files available in the working dataset; Supplementary Tables S1–S22; canonical S02/S03 polarization files; hash-verified S16-derived cycle/replicate extracts; S16b validation outputs; BEGREM MODEL complete curve-level predictions, paired ΔRMSE and AICc records, residual and parameter diagnostics, and LOSO outputs; executable model/reanalysis/reconstruction scripts; frozen analysis protocols; and SHA-256 manifests. The original large S16/S16b vendor workbooks remain in their authoritative public repositories and are linked through the supplied acquisition/provenance records and source hashes.

## Supplementary Materials

The following supporting information can be downloaded at: https://www.mdpi.com/article/10.3390/microorganisms14102187/s1, Supplementary Table S1 (source-data manifest), Table S2 (curated study-level database), Table S3 (harmonized variable dictionary), Tables S4 and S4b (parameter bounds and seed/context values), Table S5 (curve-level quality-control records), Table S6 (archived model-comparison outputs), Table S7 (reanalysis summary), Table S8 (classical model reconstruction), Table S9 (classical RMSE uncertainty/AICc audit), Table S10 (ORP-substrate identifiability), Table S11 ($Q_{m}$ empirical/structural identifiability), Table S12 (uncertainty and analysis-availability matrix), Table S13 (model-closure specification), Table S14 (empirical $Q_{m}$ identification from S16), Table S15 (independent frozen-parameter validation in S16b), Table S16 (common-shape S02 ↔ S03 LOSO), Table S17 (BEGREM MODEL complete provenance and reconstruction map), Table S18 (de novo 99-curve full/parsimonious reconstruction), Table S19 (reconstructed full-layer S02 ↔ S03 LOSO), Table S20 (reconstructed robustness and uncertainty summary), Table S21 (curve-level held-out residual diagnostics), and Table S22 (reconstruction-boundary and influence audit). The archive also contains point-level predictions, complete paired ΔRMSE and AICc records, reconstructed parameter diagnostics, frozen protocols, executable scripts, source-data provenance, and SHA-256 integrity manifests. Supplementary Figure S1 (model median reconstruction); Supplementary Figure S2 (paired exact-comparator reconstruction); Supplementary Figure S3 (exact-reconstructable ΔAICc distribution); Supplementary Figure S4 (full-BEGREM S02 ↔ S03 LOSO); Supplementary Figure S5 (full versus parsimonious BEGREM reconstruction); and Supplementary Figure S6 (full-BEGREM held-out RMSE distribution) are included in the reconstruction-analysis directory. The archive additionally retains the underlying raw and derived data files, curve-level predictions and diagnostics, frozen analysis protocols, executable reproducibility scripts, source-data provenance records, and SHA-256 integrity manifests corresponding to the analyses reported in the manuscript.

## Abbreviations

DET	direct extracellular electron transfer
EET	extracellular electron transfer
EIS	electrochemical impedance spectroscopy
MET	mediated extracellular electron transfer
MFC	microbial fuel cell
AICc	corrected Akaike information criterion
AOC	activation-Ohmic-concentration model
BEGREM	mechanistic bioelectrochemical model coupling gut microbial metabolism
BES	bioelectrochemical system
CV	cyclic voltammetry
OCP	open-circuit potential
ORP	oxidation-reduction potential
Q_m_	effective microbial reducing-equivalent reservoir
RMSE	root mean square error
SCFA	short-chain fatty acid
